# Physical and Mechanical Properties of 3D-Printed Provisional Crowns and Fixed Dental Prosthesis Resins Compared to CAD/CAM Milled and Conventional Provisional Resins: A Systematic Review and Meta-Analysis

**DOI:** 10.3390/polym14132691

**Published:** 2022-06-30

**Authors:** Saurabh Jain, Mohammed E. Sayed, Mallika Shetty, Saeed M. Alqahtani, Mohammed Hussain Dafer Al Wadei, Shilpi Gilra Gupta, Ahlam Abdulsalam Ahmed Othman, Abdulkarim Hussain Alshehri, Hatem Alqarni, Abdulaziz Hussain Mobarki, Khalid Motlaq, Haifa F. Bakmani, Asma A. Zain, Abdullah J. Hakami, Moayad F. Sheayria

**Affiliations:** 1 Department of Prosthetic Dental Sciences, College of Dentistry, Jazan University, Jazan 45142, Saudi Arabia; ahalshehri@jazanu.edu.sa; 2 Rutgers School of Dental Medicine, Rutgers University, Newark, NJ 07103, USA; 3 Department of Prosthodontics, Yenepoya Dental College, Mangaluru 575018, India; mallikashetty@yenepoya.edu.in; 4 Department of Prosthetic Dentistry, College of Dentistry, King Khalid University, Abha 62529, Saudi Arabia; smaalqahtani@kku.edu.sa; 5 Department of Restorative Dental Science, College of Dentistry, King Khalid University, Abha 62529, Saudi Arabia; moalwadai@kku.edu.sa; 6 Department of Prosthodontics, Government College of Dentistry, Indore 452001, India; shilpigilra@gmail.com; 7 Department of Fixed Prosthodontics, Faculty of Dentistry, Sana’a University, Sana’a 421302, Yemen; ahlam.abdulsalam@yahoo.com; 8 Restorative and Prosthetic Dental Science Department, College of Dentistry, King Saud Bin Abdulaziz University for Health Sciences, King Abdullah International Medical Research Center, Riyadh 14611, Saudi Arabia; qarnih@ksau-hs.edu.sa; 9 Department of Prosthodontics Dentistry, Ministry of Health Saudi Arabia, Jazan 82511, Saudi Arabia; abdalazizhussain@gmail.com; 10 Restorative Department, Faculty of Dentistry, King Khalid University, Abha 62529, Saudi Arabia; dr.almotlaq5055@gmail.com; 11 College of Dentistry, Jazan University, Jazan 45142, Saudi Arabia; haifabook19@gmail.com (H.F.B.); asmazain112@gmail.com (A.A.Z.); abdullah.dentist17@gmail.com (A.J.H.); 12 Private Dental Practice, Jeddah 22361, Saudi Arabia; moayadshearia@gmail.com

**Keywords:** provisional dental resins, PMMA, 3D printing, CAD/CAM, provisional crowns, provisional fixed dental prosthesis, mechanical properties, physical properties, fracture strength, color stability, surface roughness, wear resistance, flexural strength, water absorption and solubility, modulus of elasticity, peak stress

## Abstract

Newly introduced provisional crowns and fixed dental prostheses (FDP) materials should exhibit good physical and mechanical properties necessary to serve the purpose of their fabrication. The aim of this systematic literature review and meta-analysis is to evaluate the articles comparing the physical and mechanical properties of 3D-printed provisional crown and FDP resin materials with CAD/CAM (Computer-Aided Designing/Computer-Aided Manufacturing) milled and conventional provisional resins. Indexed English literature up to April 2022 was systematically searched for articles using the following electronic databases: MEDLINE-PubMed, Web of Science (core collection), Scopus, and the Cochrane library. This systematic review was structured based on the guidelines given by the Preferred Reporting Items for Systematic Reviews and Meta-Analyses (PRISMA). The focused PICO/PECO (Participant, Intervention/exposure, Comparison, Outcome) question was: ‘Do 3D-printed (P) provisional crowns and FDPs (I) have similar physical and mechanical properties (O) when compared to CAD/CAM milled and other conventionally fabricated ones (C)’. Out of eight hundred and ninety-six titles, which were recognized after a primary search, twenty-five articles were included in the qualitative analysis, and their quality analysis was performed using the modified CONSORT scale. Due to the heterogeneity of the studies, only twelve articles were included for quantitative analysis. Within the limitations of this study, it can be concluded that 3D-printed provisional crown and FDP resin materials have superior mechanical properties but inferior physical properties compared to CAD/CAM milled and other conventionally fabricated ones. Three-dimensionally printed provisional crowns and FDP materials can be used as an alternative to conventional and CAD/CAM milled long-term provisional materials.

## 1. Introduction

A well-fabricated provisional crown or fixed dental prosthesis (FDP) is vital in achieving a good-quality definitive prosthesis. A provisional crown or FDP must maintain the tooth position, protect the pulp, maintain the periodontal relationship, and establish function and aesthetics [1,2,3,4]. In clinical scenarios where provisionalization is required for longer durations (dental implant therapy or in full mouth rehabilitation cases involving extensive occlusal reconstruction), provisional restorative materials should have good physical and mechanical properties to avoid failures under prolonged functional loading [5,6,7].

Based on the composition, provisional restoration materials can be broadly divided into two types: (a) polymethylmethacrylate (PMMA) or polyethyl methacrylate (PEMA) based and (b) bis-acrylic or dimethacrylates resins [8]. PMMA was first used as a provisional material, and with the advancements in material science, newer materials such as bis-acrylics were introduced to provide the best clinical outcomes [8,9]. These materials were used using conventional techniques, which can be direct, indirect, or a combination of both [10].

The introduction of digital technology (computer-aided designing and computer-aided manufacturing (CAD/CAM)) in the field of prosthodontics has revolutionized the methods of providing treatments to patients. The CAD/CAM milling or subtractive manufacturing technique uses pre-polymerized resin blocks milled to provide the desired shape [11,12,13,14]. Multiple studies have compared the physical and mechanical properties of CAD/CAM milled provisional resins with conventional provisional resins and found them to have superior properties [1,4,15,16]. In addition, the inherent problems of conventional PMMA-based provisional materials (high polymerization shrinkage, high residual monomer) were minimized by using subtractive manufacturing techniques [17,18,19,20,21]. A recent literature review by Batisse et al. [21] compared the CAD/CAM and conventional denture base resins and concluded that CAD/CAM denture base resins have better physical and mechanical properties than conventional denture base resins.

More recently, additive manufacturing/three-dimensional (3D) printing techniques have gained popularity. This technique fabricates the desired prosthesis by adding small parts of the material layer by layer [22,23]. The 3D printing methods include Stereolithography (SLA), Digital light processing (DLP), Selective Laser Sintering (SLS), and Fused Deposition Modelling (FDM) [24].

Compared to CAD/CAM milling, the 3D printing technique reduces the manufacturing time and causes less wastage of raw material; thus, it can be a cost-effective option for fabricating provisional crowns and FDPs [25]. Studies comparing the physical and mechanical properties of provisional 3D-printed resins (used for fabricating provisional crowns and FDPs) with conventional and CAD/CAM milled provisional resins have shown varied results [16,26,27,28,29,30,31].

There is no known systematic review that assesses the mechanical and physical properties of 3D-printed provisional resins compared to CAD/CAM milled and conventional resins. These outcomes are important, as they can help select the best materials and techniques for fabricating provisional crowns and FDPs. Thus, the aim of this systematic literature review and meta-analysis is to compare and analyze the articles comparing the physical and mechanical properties of 3D-printed provisional crown and FDP resin materials with CAD/CAM milled and conventional provisional resins. The null hypotheses framed are that there are no differences in physical and mechanical properties of 3D-printed provisional crowns and FDP resins when compared to conventional and CAD/CAM milled provisional resins.

## 2. Materials and Methods

This systematic review was structured based on the guidelines given by the Preferred Reporting Items for Systematic Reviews and Meta-Analyses (PRISMA) (Supplementary Materials) [32]. The study was pre-registered on the PROSPERO registration platform (No.: 338845).

### 2.1. Selection Criteria

Inclusion and exclusion criteria are listed in Table 1.

### 2.2. Exposure and Outcome

The focused PICO/PECO (Participant, Intervention/exposure, Comparison, Outcome) question was: ‘Do 3D-printed (I) provisional crowns and FDPs (P) have similar physical and/or mechanical properties (O) when compared to CAD/CAM milled and other conventionally fabricated ones (C)’:

1. P—Provisional Crowns and Fixed Dental Prosthesis;
2. I—3D-Printed Technique;
3. C—CAD/CAM or Conventional Technique;
4. O—Physical/Mechanical Properties.

### 2.3. Information Sources and Search Strategy

Two independent authors (S.J. and M.S.) systematically searched the indexed English literature using the following electronic databases: MEDLINE-PubMed, Web of Science (core collection), Scopus, and the Cochrane library. The search for the articles was performed in February 2022, and then it was updated in April 2022. Combinations of Medical subject heading terms (MeSH) and Non-MeSH terms along with Boolean operators were used to perform the search. Details of search strings used for the systematic search are mentioned in Table 2. Reference lists of the relevant articles were screened manually for supplementary pertinent articles which were not detected during the electronic search. The search strategy was modified according to the requirements of the database searched.

### 2.4. Study Selection and Data Extraction

Duplicate articles were removed. The titles and abstracts of the identified articles were screened based on the pre-set inclusion and exclusion criteria (by S.G.G. and M.E.S.). Later, S.J. and M.S. cross-checked the shortlisted articles after reviewing the full texts, and disagreements related to conflicting articles were resolved after a discussion between the four authors (S.J., M.S., M.E.S., S.G.G.). S.J., M.S., M.E.S., A.A.A.O., and S.M.A. used self-designed tables to tabulate the relevant data. The information extracted was divided into two categories; Table 3 was a common table for all the selected articles giving information about the author’s name, year of publication, study type, studied characteristic and property, sample size, trade name and main composition of the evaluated materials, specimen fabrication technique, shape and dimensions of the tested samples, and layer thickness and orientation of the 3D-printing. Quality analysis results of the included studies are listed in Table 4. Moreover, Table 5, Table 6, Table 7, Table 8, Table 9, Table 10, Table 11, Table 12, Table 13, Table 14 and Table 15 gave comprehensive information about each physical or mechanical property tested. Details in these tables were related to the exposure agent/aging technique, testing machine, results of the property tested for each type of material, and authors’ conclusions and suggestions.

### 2.5. Quality Assessment of Included Studies

As all the selected studies were in vitro studies, so the Modified CONSORT scale for in vitro studies given by Faggion C. [33,34] was used to assess the quality of the included studies. The fourteen items included in this scale were as follows: Item 1: Structured abstract. Items 2a and 2b are related to the introduction. Item 2a: scientific background and explanation of rationale; Item 2b: Introduction should have specific objectives and/or hypotheses). Items 3 to 10 are related to Methodology. Item 3: intervention for each group; Item 4: Completely defined, pre-specified primary, and secondary measures of outcome; Item 5: sample size determination; Item 6: Method used to generate the random allocation sequence; Item 7: Mechanism used to implement the random allocation sequence; Item 8: Who generated the random allocation sequence; Item 9: If done, who was blinded after assignment to intervention and how; Item 10: Statistical methods used to compare groups for primary and secondary outcomes; Item 11: For each primary and secondary outcome, results for each group and the estimated size of the effect and its precision (for example 95% confidence interval); Item 12: Trial limitations; Item 13: Sources of funding and other support, role of funders; Item 14: Where the full trial protocol can be accessed, if available (Table 4).

### 2.6. Quantitative Assessment

Review Manager 5.4.1 was used to perform a Meta-analysis in Non-Cochrane Review mode [35]. Since all the physical and mechanical properties were measured and reported in studies on a continuous scale, inverse variance was used as the statistical method. The fixed-effect model was used under the assumption that all effect estimates are estimating the same underlying intervention effect. Since the measurement tools and scales varied among different studies, standardized mean difference was used. A 95% confidence interval was used to express the results of individual studies and the pooled result. Chi-square was used to measure heterogeneity, and a *p*-value < 0.05 was considered significant. I^2^ was also calculated and reported in the results. Statistical significance was calculated for the overall effect; if *p* was less than 0.05, the null hypothesis was rejected.

**Table 3:** Summary of the studies included in the systematic review.

Author and Year	Study Type	Studied Characteristics	Studied Property	Sample Size (*n*)	Trade Name and Manufacturer of the Evaluated Materials	Main Chemical Composition	Specimen Fabrication Technique	Shape and Dimension of Tested Resins Samples	Layer Thickness and Orientation of Printing
Digholkar et al., 2016 [36]	In vitro	Flexural strength Microhardness	MP	*n* = 60 (20 per group)	(A) Heat-activated PMMA (N/M) (B) Ceramill TEMP (AmannGirrbach) (C) E-Dent 100 (Envisiontec GmbH)	(A) Heat cure PMMA (B) PMMA (C) Tetrahydrofurfuryl methacrylate	(A) Conventional (B) CAD/CAM Milled (C) 3D-Printed	Bars (25 mm × 2 mm × 2 mm)	layer thickness: N/M Orientation: N/M
Tahayeri et al., 2018 [37]	In Vitro	Elastic modulus Peak stress	MP	N/M	(A) Jet (Lang Dental In(C) (B) Integrity (Dentsply) (C) NextDent C&B resin (NextDent)	(A) PMMA (B) Bisacrylic (C) Methacrylic oligomers	(A) and (B) Conventional self-cure (C) 3D-printed	Bars (25 × 2 × 2 mm)	layer thickness: 100 μm Orientation: 90°
Park et al., 2018 [26]	In vitro	Wear resistance	MP	*n* = 60 (20 per group)	(A) Jet (Lang Dental Mfg. Co.) (B) Vipiblock PMMA Monocolor (VIPI) (C) C&B NextDent (NextDent) (PMM(A)	(A) PMMA (B) PMMA (C) PMMA	(A) conventional self-care (B) CAD/CAM milled (C) 3D printing	Rectangular parallelepipeds (15 × 10 × 10 mm)	layer thickness: 100 μm Orientation: 0°
Kessler et al., 2019 [27]	In Vitro	Three-body wear	MP	*n* = 40 (8 per group)	(A) TetricEvoCeram (Vivadent) (B) Telio CAD (Ivoclar) (C) 3Delta temp (Deltame(D) (D) Nextdent C&B (NextDent) (E) Freeprint temp (Detax)	(A) Bis-GMA (B) PMMA (C) Methacrylate (D) Methacrylic oligomers (E) Methacrylate-based resins	(A) Conventional (B) CAD/CAM Milling (C), (D), and (E) 3D-printing	Wheel-shaped	layer thickness: N/M Orientation: N/M
Reeponmaha et al., 2020 [16]	In vitro	Fracture Strength	MP	*n* = 40 (10 per group)	(A) Unifast Trad (GC chemicals) (B) Protemp 4 (3 M ESP(E) (C) Brylic Solid (Sagemax bioceramics) (D) Freeprint Temp (Detax GmbH)	(A) Methylmethacrylate resin (B) Bis-acryl resin (C) Highly polymerized PMMA resin (D) Photopolymerized Methacrylate-based resins	((A) and ((B): Conventional (C) CAD/CAM Milling (D) 3D-printing	Provisional crowns cemented on prepared epoxy die replicated from prepared tooth	layer thickness: N/M Orientation: N/M
Ibrahim et al., 2020 [38]	In vitro	Fracture Resistance	MP	*n* = 16 (8 per group)	(A) Telio CAD disc ( Ivoclar Vivadent) (B) NextDent C&B resin (NextDent B.V)	(A) PMMA (B) MMA	(A) CAD/CAM Milling (B) 3D-printing	Provisional crowns cemented on prepared epoxy die replicated from prepared tooth	layer thickness: 50 μm Orientation: N/M
Shin et al., 2020 [28]	In vitro	Color stability Water sorption and solubility	PP	*n* = 200 (40 per group)	(A) Polycarbonate block (Line dental la(B) (B) Vipi block monocolor (Dental VIPI Ltd.) (C) MAZIC Duro (Vericom) (D) Nextdent C&B (Nextdent) (E) denture teeth A2 resins (Formlabs In(C)	(A) Polycarbonate (B) PMMA (C) dispersed-filler composite (DF(C) (D) Methacrylic oligomers (E) UDMA	(A), (B), (C): CAD/CAM Milling (D), (E) 3D-printing	disk-shaped (10 mm diameter, 3 mm thickness)	layer thickness: 100 μm Orientation: N/M
Suralik et al., 2020 [39]	In vitro	Fracture Strength	MP	*n* = 45 (15 per group)	(A) Jet (Lang Dental Inc.) (B) Zirlux Temp (Henry Schein) (C) Freeprint Temp (DETAX GmbH)	(A) PMMA (B) PMMA (C) Methacrylate-based resins	(A) Conventional (Self-cur(E) (B) CAD/CAM Milling (C) 3D-Printing	Provisional 3-unit fixed dental prosthesis (FDP) attached to implant abutments of the master metal typodont, with no luting agent.	layer thickness: 50 μm Orientation: 0°
Reymus et al., 2020 [40]	In vitro	Fracture load	MP	*n* = 195 (15 per group)	(A) Luxatemp (DMG) (B) Telio CAD (Ivoclar-Vivadent) (C) Experimental (GC Europ(E) (D) NextDent C&B (NextDent) (E) Freeprint temp (Detax) F) 3Delta temp (Deltame(D)	(A) Bis-acryl Methacrylate (B) PMMA Polymer (C) Methylmethacrylates (D) Methylmethacrylates (E) Methylmethacrylates F) Methylmethacrylates	(A) Conventional (B) CAD/CAM milling (C), (D), (E), and (F): 3 D printing	A full-anatomic three-unit FDP attached to a steel abutment model with no luting agent.	layer thickness: N/M Orientation: N/M Long-axis positioned either occlusal, buccal, or distal to the printer’s platform.
Revilla-León et al., 2020 [41]	In vitro	Color dimensions	PP	*n* = 420 (60 per group)	(A) Protemp 4 (3M ESP(E) (B) Anaxdent (Anaxdent) (C) FreePrint Temp (Detax) (D) E-Dent 400 (EnvisionTE(C) (E) C&B (NextDent) (F) C&B MFH ((NextDent) (G) VeroGlaze MED620 (Stratasys)	(A) Bis-acryl composite (B) PMMA (C) Monomer-based acrylic esters (D) Monomer based on acrylic esters (E) Methylmethacrylates F) Microfilled hybrid G) Monomer based on acrylic esters	(A) and (B) Conventional (C), (D), (E), and (F): 3D-printed	Discs (10 mm diameter, 2 mm thickness)	layer thickness: N/M Orientation: N/M
Atria et al., 2020 [42]	In vitro	Color stability Surface roughness	PP MP	*n* = 40 (10 per group)	(A) Marche (March(E) (B) Protemp (3M ESP(E) (C) Telio CAD (Ivoclar Vivadent) (D) Raydent C&B (3D-Materials)	(A) acrylic resin (B) bis-acryl resin (C) PMMA (D) Hybrid composite Resin	(A) and (B): Conventional (C) CAD/CAM Milled (D) 3D-Printed	Rectangular blocks (1 mm × 1.7 mm × 0.6 and 1.3-mm thickness)	layer thickness: 100 μm Orientation: 90°
Park et al., 2020 [43]	In vitro	Flexural strength	MP	*n* = 75 (15 per group)	(A) Jet Tooth ShadeTM Powder (Lang Dental Co.) (B) ViPi (VIPI Co.) (C) NextDent C&B (NextDent Co.) (D) Standard (GPGR04) (Formlabs Co.) (E) PLA (ColorFabb Co.)	(A) PMMA (B) PMMA (C) PMMA (D) PPMA (E) Polylactic acid	(A) Conventional (B) CAD/CAM Milled (C) 3D-Print: DLP (D) 3D-print: SLA (E) 3D-print: FDP	3-unit FDP fitted on the abutment of the metal jig without cementation	layer thickness: (C) & (D) 25–100 um (E) 100–500 um Orientation: 30°
Song et al., 2020 [44]	In vitro	color stability Water sorption & Solubility	PP	For water sorption and solubility: *n* = 60 (10 per group) For Color stability: *n* = 120 (20 per group, 10 for coffee and 10 for te(A)	(A) Alike (GC Co.) (B) Luxatemp Automix plus (DMG) (C) PMMA Disk (Yamahachi Dental Co) (D) Telio CAD (Ivoclar Vivadent) (E) VeroGlaze (Stratasys) (F) E-dent 100 (EnvisionTEC GmbH)	(A) Polymethyl methacrylate (B) Bis-acryl methacrylate (C) Polymethyl methacrylate (D) Polymethyl methacrylate (E) Bio-compatible photopolymer (F) Multifunctional Acrylic resin	(A) and (B) Conventional (C) and (D): CAD/CAM Milled (E) and (F): 3D-printed	disk-shaped (15 mm diameter, 1 mm thickness)	layer thickness: N/M Orientation: N/M
Yao et al., 2021 [45]	In vitro	color stability	PP	*n* = 80 (40 per group)	(A) Temp Esthetic 98 (Harvest Dental Products) (B) NextDent Crown and Bridge resin (NextDent)	(A) PMMA (B) Methylmethacrylates	(A) CAD/CAM milling (B) 3D-Printing	Provisional crowns cemented to the 3D-printed abutment teeth with interim luting agent	layer thickness: N/M Orientation: N/M
Abad-Coronel et al., 2021 [46]	In vitro	Fracture Resistance	MP	*n* = 40 (20 per group)	(A) Vipiblock Trilux: (VIPI) (B) PriZma 3D Bio Prov: (MarkertechLabs)	(A) PMMA (B) Light-Curing Micro Hybrid Resin	(A) CAD/CAM milling (B) 3D-Printing	A 3-unit FDP fitted on a 3D-printed resin master typodont without any fixing agent.	layer thickness: N/M Orientation: N/M
Myagmar et al., 2021 [47]	In vitro	Wear resistance Surface roughness	MP	*n* = 48 (16 per group, later divided into 8 per subgroup based on cycles of chewing simulation)	(A) JetTM (Lang Dental Manufacturing) (B) Yamahachi PMMA Disk (Yamahachi Dental Manufacturing) (C) NextDent C&B (NextDent)	(A) PMMA (B) PMMA (C) Methacrylic oligomers	(A) Conventional (B) CAD/CAM Milled (C) 3D-Printed	rectangular parallelepipeds (15 × 10 × 10 mm)	layer thickness: 100 μm Orientation: 0°
Tas¸ın et al., 2021 [48]	In vitro	color stability Surface roughness	PP MP	*n* = 320 (80 per group) Divided into 2 subgroups n = 40 (i) conventional polishing (ii) surface sealant covering each group (*n* = 10) immersed in 4 different solutions	(A) Temdent Classic (Schütz-Dental) (B) Protemp 4 (3M ESP(E) (C) Duo Cad (FSM DENTAL) (D) Temporis (DWS)	(A) PMMA (B) Bis-acryl composite resin (C) PMMA (D) Hybrid composite Resin	(A) and (B) Conventional (C) CAD/CAM Milled (D) 3D-printed	disk-shaped (10 mm diameter, 2 mm thickness)	layer thickness: 100 μm Orientation: N/M
Revilla-León et al., 2021 [49]	In vitro	Knoop hardness	MP	*n* = 360 (60 per group) *n* = 20 per group used for testing each property	(A) Protemp 4 (3M ESP(E) (B) Anaxdent new outline dentin (Anaxdent) (C) FreePrint temp (Detax) (D) E-Dent 400 C&B MFH (Envisionte(C) (E) NextDent C&B MFH (3D Systems) (F) Med620 VEROGlaze (Stratasys)	(A) bis-acryl resin (B) acrylic resin (C)Methylmethacrylates (D) Monomer based on acrylic esters (E) Micro-Filled Hybrid Methacrylic oligomers (F) N/M	(A) and (B): Conventional (C), (D), (E), and (F): 3D-Printed	Disks (10 mm diameter, 2 mm thickness)	layer thickness: 50 μm Orientation: 90°
Mayer et al., 2020 [50]	In vitro	Fracture load & Two-body wear	MP	*n* = 152 (48 per group for 3D-printed and 8 for CAD/CAM Mille(D)	((A) Telio CAD disc (Ivoclar Vivadent) (B) Freeprint temp (Detax) ((C) GC Temp PRINT (GC Europe) ((D) Next dent C&B MFH (NextDent) After printing, excessive resin removed from the specimen’s surface in 3 ways: (i) Centrifugation (CEN); (ii) Chemical cleaning by Isopropanol (ISO); (iii) Chemical cleaning by Yellow Magic (YEL)	(A) PMMA (B) Methylmethacrylates (C) UDMA (D) Methylmethacrylates	(A) CAD/CAM milling (B), (C), and (D): 3D-Printing	A full anatomic, three-unit FDP fixed on steel abutment model with a dual-cure self-adhesive resin composite cement	layer thickness: N/M Orientation: N/M
Henderson et al., 2022 [51]	In vitro	Failure Load	MP	*n* = 180 (60 per group) Storage time: 1 day and 30 days & Loading rate: 1, 10 and Combined 1 and 10 mm/min	(A) 3M-Paradigm (3M Oral Car(E) (B) Solid Shade PMMA Disc (TD Dental Supply) (C) Dentca Crown and Bridge resin (Dentc(A)	(A) Bis-acryl resin (B) PMMA (C) bis-acryl resin	(A) Conventional (B) CAD/CAM milling (C) 3D-Printing	3-unit interim FDP cemented onto 3D-printed resin dies.	layer thickness: N/M Orientation: N/M
Martín-Ortega et al., 2022 [52]	In vitro	Fracture Resistance	MP	*n* = 40 (10 per group) (10 each anterior and posterior, CAD/CAM milled and 3D-printe(D)	(A) and (C): Vivodent CAD Multi: (Ivoclar Vivadent AG) (B) and (D): SHERAprint-cb (Sher(A)	(A) PMMA (B) Photopolymer interim dental resin	(A) CAD/CAM milling (B) 3D-Printing	Full anatomic crowns (20 anterior and 20 posterior) cemented on implant abutment with autopolymerizing composite resin cement	layer thickness: 50 μm Orientation: 45°
Simoneti et al., 2022 [53]	In vitro study	flexural strength Vickers microhardness Elastic Modulus surface roughness before and after polishing	MP	Interim single crowns *n* = 40 (10 per group) Rectangular blocks *n* = 40 (10 per group) disks *n* = 40 (10 per group)	(A) Dencor (Artigos Odontológicos Clássico Ltd.(A) (B) Yprov Bisacryl (Yller Biomaterials) (C) PA2201 (Stratasys Direct Manufacturing) (D) Gray Resin (Formlabs In(C)	(A) PMMA (B) Bis-acryl resin (C) PMMA (D) Oligomers methacrylates	(A) and (B): Conventional (C) and (D) 3D-Printed SLS & SLA	Interim single crowns rectangular blocks 4 × 2 × 10 mm disks 10 mm diameter, 2 mm thickness	layer thickness: N/M Orientation: N/M
Crenn et al., 2022 [29]	In vitro	3-point bending test (elastic modulus) Flexural strength Hardness	MP	*n* = 40 (10 per group)	(A) Integrity (Dentsply Caulk) (B) Unifast (GC, Tokyo) (C) PLA Bio source (Nanovi(A) (D) Temporary CB (Formlabs)	(A) Bisacrylic (B) Methylmethacrylate resin (C) Polylactic acid (D) Esterification products of 4,4′-isopropylidenediphenol	(A) and (B): Conventional (C) 3D-printed (FDM) (D) 3D-Printed (SL(A)	Bars (25 mm × 2 mm × 2 mm)	layer thickness: FDM: 100 μm SLA: 50 μm Orientation: FDM: 0° SLA: 0°
Tas¸ın et al., 2022 [30]	In vitro	Flexural strength Resilience Toughness Modulus of elasticity	MP	*n* =120 (30 per group, 10 each for flexural strength, resilience, and toughness) Sub group (*n* = 10) based on different thermocycling	(A) Temdent Classic (Schütz-Dental) (B) Protemp 4 (3M ESP(E) (C) Duo Cad (FSMDENTAL) (D) Temporis (DWS)	(A) MMA (B) Bis-acryl (C) PMMA (D) Composite resin	(A) and (B): conventional (C) CAD/CAM Milled (D) 3D-printed	Rectangular plate (25 × 2 × 2 mm)	layer thickness: 60 μm Orientation: 90°
Pantea et al., 2022 [31]	In vitro	Flexural strength Elastic Modulus	MP	*n* = 40 (10 per group, 5 each for flexural strength and compression strength)	(A) Duracyl (SpofaDental a.s) (B) Superpont C + B (SpofaDental a.s.) (C) NextDent C&B MFH (NextDent) (D) HARZ Labs Dental Sand (HARZ Labs)	(A) Auto-polymerized (PMM(A) (B) Pressure/heat-cured (PMM(A) (C) Microfilled hybrid PMMA (D) PMMA	(A) Conventional self-cure (B) Conventional heat cured (C) and (D): 3D-Printed	For Flexural strength: Bar shaped (80 × 20 × 5 mm) For Compressive strength: Cylindrical shaped (25 × 25 mm)	layer thickness: 50 μm Orientation: N/M

MP: Mechanical Property; PP: Physical Property; FS: Fracture Strength; FR: Fracture resistance; FL: Fracture load; FaL: Failure Load; N/M: Not Mentioned; CAD/CAM: Computer-Aided Designing/Computer-Aided Manufacturing; FDP: Fixed Dental Prosthesis; SLA: Stereolithography; SLS: Selective laser sintering; FDM: Fused deposition modeling; DLP: Digital light processing; UDMA: urethane dimethacrylate.

**Table 4:** Quality analysis results of the included studies.

Item	1	2a	2b	3	4	5	6	7	8	9	10	11	12	13	14
Studies															
Digholkar et al., 2016 [36]	Y	Y	Y	Y	Y	N	N	N	N	N	Y	Y	N	Y	N
Tahayeri et al., 2018 [37]	Y	Y	Y	Y	Y	N	N	N	N	N	Y	Y	N	Y	Y
Park et al., 2018 [26]	Y	Y	Y	Y	Y	N	N	N	N	N	Y	Y	N	Y	N
Kessler et al., 2019 [27]	Y	Y	Y	Y	Y	N	N	N	N	N	Y	Y	Y	Y	N
Reeponmaha et al., 2020 [16]	Y	Y	Y	Y	Y	Y	Y	N	N	N	Y	Y	N	Y	N
Ibrahim et al., 2020 [38]	Y	Y	Y	Y	Y	N	N	N	N	N	Y	Y	N	N	N
Shin et al., 2020 [28]	Y	Y	Y	Y	Y	N	Y	Y	N	N	Y	Y	Y	Y	Y
Suralik et al., 2020 [39]	Y	Y	Y	Y	Y	N	N	N	N	N	Y	Y	Y	Y	Y
Reymus et al., 2020 [40]	Y	Y	Y	Y	Y	N	N	N	N	N	Y	Y	Y	Y	N
Revilla-León et al., 2020 [41]	Y	Y	Y	Y	Y	Y	N	N	N	N	Y	Y	Y	N	N
Atria et al., 2020 [42]	Y	Y	Y	Y	Y	N	N	N	N	N	Y	Y	N	N	N
Park et al., 2020 [43]	Y	Y	Y	Y	Y	N	N	N	N	N	Y	Y	Y	Y	N
Song et al., 2020 [44]	Y	Y	Y	Y	Y	N	N	N	N	N	Y	Y	N	N	N
Yao et al., 2021 [45]	Y	Y	Y	Y	Y	N	N	N	N	N	Y	Y	Y	N	N
Abad-Coronel et al., 2021 [46]	Y	Y	Y	Y	Y	N	N	N	N	N	Y	Y	Y	Y	Y
Myagmar et al., 2021 [47]	Y	Y	Y	Y	Y	N	N	N	N	N	Y	Y	Y	Y	N
Taşın et al., 2021 [48]	Y	Y	Y	Y	Y	Y	N	N	N	N	Y	Y	Y	N	N
Revilla-León et al., 2021 [49]	Y	Y	Y	Y	Y	Y	Y	N	N	N	Y	Y	Y	Y	N
Mayer et al., 2021 [50]	Y	Y	Y	Y	Y	N	N	N	N	N	Y	Y	N	N	N
Henderson et al., 2021 [51]	Y	Y	Y	Y	Y	N	N	N	N	N	Y	Y	N	N	N
Martín-Ortega et al., 2022 [52]	Y	Y	Y	Y	Y	N	Y	N	N	N	Y	Y	Y	N	N
Simoneti et al., 2022 [53]	Y	Y	Y	Y	Y	N	N	N	N	N	Y	Y	Y	Y	N
Crenn et al., 2022 [29]	Y	Y	Y	Y	Y	Y	N	N	N	N	Y	Y	N	Y	N
Taşın et al., 2022 [30]	Y	Y	Y	Y	Y	Y	N	N	N	N	Y	Y	Y	N	N
Pantea M. et al., 2022 [31]	Y	Y	Y	Y	Y	N	N	N	N	N	Y	Y	Y	Y	Y

**Table 5:** Color change (ΔE/ΔE_00_) Results.

Author and Year	Immersion Media/Surface Treatment	Immersion/Exposure Duration/Aging	Mean Change in Color of Conventional Polymerized Resin	Mean Change in Color of CAD/CAM Milled Provisional Resin	Mean Change in Color of 3D-Printed Provisional Resin	Instrument Used	Authors Suggestions/Conclusions
Yao et al., 2021 [45]	(i) Control (no surface treatment) (ii) Polishing (iii) Polishing + Optiglaze coating (iv) Polishing + Skinglaze coating	Aging: Thermocycling: 5000 cycles at 5–50 °C (simulating 6 months of physiological aging)	N/A	ΔE (i) 2.38 ± 0.44 (ii) 1.83 ± 0.51 (iii) 1.01 ± 0.38 (iv) 1.85 ± 0.78	ΔE (i) 3.83 ± 0.71 (ii) 2.66 ± 0.89 (iii) 1.37 ± 0.67 (iv) 1.40 ± 0.73	Digital spectrophotometer (Vita Easyshade V)	ΔE: 3D-Printed PMMA > CAD/CAM Milled PMMA Surface coating reduces the change in color
Shin et al., 2020 [28]	Immersion media: (i) Grape juice (ii) Coffee (iii) Curry (iv) Distilled water	Upto 30 days inside a 37 °C (simulating 2.5 years)	N/A	ΔE_00_ Between 0.64 and 4.12	ΔE_00_ Between 4.47 and 22.85	colorimeter (Minolta Cr321 Chromameter)	ΔE_00:_ 3D-printed resins MMA > CAD/CAM milled PMMA & polycarbonate resins. For 3D-printing resins: ΔE_00_ above the clinical limit (2.25) following storage in all experimental groups.
Song et al., 2020 [44]	Immersion media: (i) Coffee (ii) Black tea	Week: 1,2,4,8,12	ΔE after week 12 (A) Alike: 9.89 ± 1.95 (coffee) 14.69 ± 3.05 (Black Tea) (B) Luxatemp Automix plus: 4.20 ± 1.57 (coffee) 6.52 ± 2.50 (Black Tea)	ΔE after week 12 (C) PMMA Disk: 10.35 ± 1.14 (coffe(E) 16.66 ± 3.05 (Black Tea) (D) Telio CAD: 21.07 ± 2.86 (coffee) 24.60 ± 4.30 (Black Tea)	ΔE after week 12 (E) VeroGlaze: 19.80 ± 2.85 (coffe(E) 16.90 ± 2.20 (Black Tea) (F) E-dent 100: 20.01 ± 3.00 (coffee) 22.13 ± 3.51 (Black Tea)	spectrocolorimeter (Xrite Benchtop Spectrophotometer)	ΔE: Telio CAD (CAD/CAM) PMMA > 3D-Printed Photopolymer & acrylic resin > PMMA Disk (CAD/CAM) > Conventional PMMA and Bisacrylic Visually perceptible color difference value (Δ(E) was demonstrated regardless of the materials and solutions.
Taşın et al., 2021 [48]	Surface treatment: (i) conventional polishing (ii) surface sealant—biscover LV Immersion Media: (A) distilled water (B) Cola (C) Coffee (D) Red Wine	Days: 1, 7 & 30	ΔE_00_ after 30 days PT & CAT Threshold values ^##^ (A) Temdent Classic (i) Polished: Distilled water (1.87): > PT Cola (3.29), Coffee, Wine > CAT (ii) Surface sealant: Distilled water < PT Cola < CAT Coffee, Wine > CAT (B) Protemp 4 (i) Polished: Distilled water: > PT Cola, Coffee, Wine > CAT (ii) Surface sealant: Distilled water (1.34): < PT Cola (2.54) < CAT Coffee, Wine > CAT	ΔE_00_ after 30 days (C) Duo Cad: (i) Polished: Distilled water: < PT Cola < CAT Coffee, Wine > CAT (ii) Surface sealant: Distilled water: < PT Coffee (2.15) and Cola < CAT Wine > CAT	ΔE_00_ after 30 days (D) Temporis: (i) Polished: Distilled water: < PT Cola < CAT Coffee, Wine > CAT (ii) Surface sealant: Distilled water: < PT Cola < CAT Coffee, Wine > CAT	Digital spectrophotometer (VITA Easyshade; Vita Zahnfabrik)	ΔE_00_: Conventional PMMA (5.35 ± 4.08) > Conventional Bis-acrylic (2.79 ± 1.54) > 3D-Printed hybrid composite (2.61 ± 1.48) > CAD/CAM Milled PMMA (2 ± 0.10). Use of a surface sealant significantly decreased the ΔE_00_ values.
Atria et al., 2020 [42]	N/A	Aging: Thermocycling: 6000 cycles at 5–50 °C	PT and CAT Threshold values ^##^ (A) Marche: 0.6 mm thickness: ΔE_00_ > PT 1.3 mm thickness: ΔE_00_ < PT (B) Protemp: 0.6 mm thickness: ΔE_00_ > PT 1.3 mm thickness: ΔE_00_ < PT	(C) Telio CAD: 0.6 mm thickness: ΔE_00_ < PT 1.3 mm thickness: ΔE_00_ < PT	(D) Raydent C&B: 0.6 mm thickness: ΔE_00_ > CAT 1.3 mm thickness: ΔE_00_ > CAT	Spectrophotometer (VITA Easyshade; Vita Zahnfabrik)	ΔE_00_: 3D-Printed hybrid composite > Conventional acryic and bisacrylic > CAD/CAM Milled PMMA

N/A: Not Applicable; PT: perceptibility threshold; CAT: clinical acceptability threshold; ^##^: The ΔE_00_ evaluation is based on: PT set at ΔE_00_ ≤ 1.30 and the CAT set at ΔE_00_ ≤ 2.25 units.

**Table 6:** Water sorption and solubility Results.

Author and Year	Water Sorption of Conventional Cured Resin	Water Sorption of CAD/CAM Milled Resin	Water Sorption of 3D-Printed Resin	Solubility of Conventional Cured Resin	Solubility of CAD/CAM Milled Resin	Solubility of 3D-Printed Resin	Authors Suggestions/Conclusions
Shin et al., 2020 [28]	N/A	(A) Polycarbonate block: 0.43% (B) Vipi block (PMMA): 1.45% (C) MAZIC Duro (DFC): ≅0.88%	(D) Nextdent C&B: 1.04% (E) Denture teeth A2 Resin: 1.21%	N/A	(A) Polycarbonate block: 0.12% (B) Vipi block (PMM(A) ≅0.34% (C) MAZIC Duro (DFC): = 0.07%	(D) Nextdent C&B: 0.53% (E) Denture teeth A2 Resin: 0.47%	Water sorption: Conventional PMMA > 3D-Printed Denture teeth A2 Resin > 3D-Printed PMMA > Conventional Polycarbonate > Conventional DFC Water Solubility: 3D-Printed PMMA > 3D-Printed Denture teeth A2 Resin > Conventional Vipi block PMMA > Conventional DFC > conventional Polycarbonate
Song et al., 2020 [4]	(A) Alike: 32.23 ± 5.93 (B) Luxatemp Automix plus: 14.15 ± 1.30	(C) PMMA Disk: 23.16 ±1.25 (D) Telio CAD: 19.13 ± 1.41	(E) VeroGlaze: 35.02 ± 1.43 (F) E-dent 100: 20.08 ± 2.27	In μgm/mm^3^ (A) Alike: 3.54 ± 1.81 (B) Luxatemp Automix plus: 0.38 ± 0.56	In μgm/mm^3^ (C) PMMA Disk: 0.84 ± 0.61 (D) Telio CAD: 0.97 ± 0.47	In μgm/mm^3^ (E) VeroGlaze: 0.52 ± 0.80 (F) E-dent 100: 2.78 ± 1.49	Water sorption: Conventional PMMA > 3D-printed photopolymer > CAD/CAM Milled (PMMA Disk) > 3D-Printed acrylic > CAD/CAM milled PMMA > Conventional bis-acrylic. Water Solubility: Conventional PMMA & 3D-printed acrylic > 1 μg/mm^3^. For other four groups <1 μg/mm^3^.

N/A: Not Applicable.

**Table 7:** Fracture strength/Fracture Resistance/Fracture Load/Failure Load Results.

Author and Year	Exposure Agent/Aging Technique	Testing Machine Used	Mean Maximum Force at Fracture for Conventional Resin (N)	Mean Maximum Force at Fracture for CAD/CAM Milled Resin (N)	Mean Maximum Force at Fracture for 3D-Printed Resin (N)	Conclusions and/or Suggestions
Reeponmaha et al., 2020 [16]	(A) Thermal Cycling: 5000 cycles at 5–55 °C (B) Cyclic occlusal load: 100 N at 4 Hz for 100,000 cycles	Universal testing machine	(A) Unifast Trad: 657.87 ± 82.84 (B) Protemp 4: 1125.94 ± 168.07	(C) Brylic Solid: 953.60 ± 58.88	(D) Freeprint Temp: 1004.19 ± 122.18	FS: Conventionally fabricated bis-acryl > 3D-printed MMA > CAD/CAM-milled PMMA > conventionally fabricated methylmethacrylate. No significant difference of fracture strength between conventionally fabricated Bis-acryl, 3D-printed MMA, and CAD/CAM-milled PMMA.
Ibrahim et al., 2020 [38]	(A) Thermocycling: 1250 cycles at 5–55 °C (B) Mechanical aging: 50 N, 37,500 cycles	Universal testing machine	N/A	(A) TelioCAD: 933.46 ± 104.49	(B) Next dent C&B resin: 1226.48 ± 48.33	FR: 3D-printed PMMA > CAD/CAM milled MMA (significantly high)
Suralik et al., 2020 [39]	N/M	Universal Instron machine	(A) Jet:300.61 ± 98.94	(B) Zirlux Temp: 294.64 ± 60.34	(C) Freeprint Temp: 408.49 ± 132.16	Fracture strength: 3D-printed Methacrylate-based resin > CAD/CAM-milled PMMA> conventionally fabricated PMMA FS of 3D-printed resin is significantly greater.
Reymus et al., 2020 [40]	Artificial aging: stored in distilled water for 21 days at 37 °C in an incubator.	Universal testing machine	(A) Luxatemp: 551.7 ± 130	(B) Telio CAD: 881.4 ± 239.2	Depending on type of post-curing unit used: [Otoflash (OF), Printbox (PB), Labolight (LL)] (C) Experimental: LL: 585.4 ± 66.8, OF: 746.4 ± 62.1, PB: 874.3 ± 104.0 (D) NextDent C&B LL: 775.9 ± 57.6, OF: 1050.4 ± 133.3, PB: 871.5 ± 398.1 (E) Freeprint temp LL: 777.6 ± 95.9, OF: 638.0 ± 175.5, PB: 598.6 ± 170.1 (F) 3Delta temp LL: 609.6 ± 118.8, OF: 868.2 ± 139.8, PB: 678.4 ± 193.7	FL: 3D-Printed MMA > or < CAD/CAM milled MMA (based on post-curing unit use(D) > Conventional Bis-acrylic
Mayer et al., 2020 [50]	Three different cleaning methods for 3D printed specimens and chewing simulation (vertical load of 50 N and a lateral movement of 0.7 mm for 480,000 masticatory cycles)	Universal testing machine	N/A	(A) Telio CAD: 1427 ± 77	(B) Freeprint temp: 623 ± 156, 539 ± 152 & 615 ± 124 ((C) GC Temp PRINT: 878 ± 139, 796 ± 121, 831 ± 260 ((D) Next dent C&B MFH: 750 ± 156, 660 ± 198, 813 ± 157	FL: CAD/CAM Milled PMMA > 3D-Printed (MMA & UDM(A) FL amongst 3D-Printed: GC Temp PRINT > Next dent C&B MFH > Freeprint temp
Abad-Coronel et al., 2021 [46]	Thermocycling: 5000 cycles, at 5 °C and 55 °C in distilled water	Universal testing machine	N/A	(A) Vipiblock Trilux: 1663.57 ± 130.25	PriZma 3D Bio Prov: 1437.74 ± 73.41	FS: CAD/CAM Milled PMMA > 3D-Printed micro-hybrid resins
Martín-Ortega et al., 2022 [52]	Thermocycling: 525,000 cycles, at 5 °C to 55 °C	Universal testing machine	N/A	(A) and (C): Vivodent CAD Multi: Anterior group: 988.4 ± 54.8 Posterior group: 423.8 ± 68.0	(B) and (D): SHERAprint-cb: Anterior group: 636.5 ± 277.1 Posterior group: 321.3 ± 128.6 N	FR: CAD/CAM Milled PMMA > 3D-Printed photopolymer resinFR: Anterior group > Posterior group
Henderson et al., 2022 [51]	Storage time in incubator (1 day or 30 days).	Universal testing machine	3M-Paradigm: Loading Rate -Combined 1 and 10 mm/min Storage time: 1 day: 537 ± 117 N 30 Days: 572 ± 139 N	Solid Shade PMMA Disc: Loading Rate -Combined 1 and 10 mm/Min Storage time: 1 day: 683 ± 115 N 30 Days: 547 ± 92 N	Dentca Crown and Bridge resin: Loading Rate—Combined 1 and 10 mm/Min Storage time: 1 day: 522 ± 98 N 30 Days: 416 ± 109 N	FaL: CAD/CAM Milled > Conventional > 3D-Printed

N: Newton; N/A: Not Applicable; N/M: Not Mentioned; FS: Fracture Strength; FR: Fracture resistance; FL: Fracture load; FaL: Failure Load.

**Table 8:** Microhardness Test Results.

Author and Year	Mean Microhardness for Conventional Resin (Kgf/mm^2^/KHN)	Mean Microhardness for CAD/CAM Milled Resin (Kgf/mm^2^/KHN)	Mean Microhardness for 3D-Printed Resin (Kgf/mm^2^/KHN)	Surface Treatment/Exposure Agent/Ageing Technique	Testing Machine Used	Authors Suggestions/Conclusions
Simoneti et al., 2022 [53]	Vickers microhardness (A) Acrylic resin: 14.2 ± 2.6 Kgf/mm^2^ (B) Bis-acryl resin: 10.7 ± 2.2 Kgf/mm^2^	NA	Vickers microhardness (C) SLA resin 8.4 ± 0.2 Kgf/mm^2^ (D) SLS resin 10.3 ± 1.0 Kgf/mm^2^	Polished specimens	Microdurometer (FM-700; Future-Tech Corp.).	Microhardness: Conventional Acrylics > Conventional Bisacrylic > 3D-printed PMMA > 3D-printed methacrylates
Revilla-León et al., 2021 [49]	Knoop hardness (A) Protemp 4: 4.92 ± 0.36 KHN (B) Anaxdent new outline dentin: 13.35 ± 5.84	N/A	Knoop hardness (C) FreePrint temp: 12.55 ± 2.93 KHN(D) E-Dent 400 C&B MFH: 13.03 ± 3.29 KHN (E) NextDent C&B MFH: 9.91 ± 3.71 (F) Med620 VEROGlaze: 13.45 ± 2.93	N/M	Microhardness tester (MMT-X7, Matsuzawa)	Knoop hardness: 3D-Printed (group (F) > Conventional PMMA (group (B) > 3D-printed acrylic esters (group (D) > 3D-Printed MMA (group (C) > 3D-Printed PMMA (group (E) > Conventional bisacrylic (group A) 3D-Printed materials have suitable MP to be used as provisional restorations.
Digholkar et al., 2016 [33]	Knoop hardness (A) heat activated PMMA: 27.36 ± 0.535 KHN	Knoop hardness (B) Ceramill TEMP: 25.33 ± 0.900 KHN	Knoop hardness (C) E-Dent 100: 32.77 ± 1.361 KHN	N/M	Microhardness tester (Reichert Austria)	3D-printed Microhybrid filled composite >Conventional heat activated PMMA >CAD/CAM milled PMMA
Crenn et al., 2022 [29]	Vickers Microhardness (A) Integrity: 27.3 ± 1.8 HV (B) Unifast: 18.4 ± 1.2 HV	N/A	Vickers Microhardness (C) PLA Bio source: 17.5 ± 0.7 HV (D) Temporary CB: 28.9 ± 2.9 HV	Polished specimens	Vickers Microhardness tester (MH3, Mekton, Turkey)	3D-printed SLA > Conventional Bisacrylic > conventional Methylmethacrylate > 3D-Printed FDM

N/A: Not Applicable; SLA, stereo lithography; SLS, selective laser sintering; N/M: Not Mentioned.

**Table 9:** Surface roughness (SR) test results.

Author and Year	SR of Conventional Material Before Surface Treatment (Ra in μm)	SR of Conventional Material After Surface Treatment (Ra in μm)	SR of CAD/CAM Milled Materials Before Surface Treatment (Ra in μm)	SR of CAD/CAM Milled Materials after Surface Treatment (Ra in μm)	SR of 3D-Printed Materials before Surface Treatment (Ra in μm)	SR of 3D-Printed Materials after Surface Treatment (Ra in μm)	Parameters of the Clinical Simulation	Exposure Medium Causing Change in SR	Measuring Device	Authors Suggestions/Conclusions
Simoneti et al., 2022 [53]	Before polishing (A) Dencor (PMMA): 4.8 ± 0.6 (B) Yprov Bisacryl (Bis-acryl resin) 1.5 ± 0.3	After polishing (A) Dencor (PMMA): 0.9 ± 0.2 (B) Yprov Bisacryl (Bis-acryl resin) 0.7 ± 0.1	N/A	N/A	Before polishing (C) PA2201 (SLS resin) 6.2 ± 0.6 (D) Gray Resin (SLA resin) 1.5 ± 0.4	After polishing (C) PA2201 (SLS resin) 1.2 ± 0.3 (D) Gray Resin (SLA resin) 0.7 ± 0.1	Polishing	N/A	Contact profiler (SJ-201; MitutoyoInc)	Ra after polishing: 3D-Printed SLS > conventional PMMA > Conventional bisacrylic = 3D printed SLA Significant reduction in SR after polishing.
Tas¸ın et al., 2021 [48]	Polishing (A) Temdent Classic (PMMA): 0.52 ± 0.09 (B) Protemp 4 (Bis-acrylic): 0.31 ± 0.04	Polishing + Surface Sealant (A) Temdent Classic (PMMA): 0.43 ± 0.07 (B) Protemp 4 (Bis-acrylic): 0.29 ± 0.05	Polishing (C) Duo Cad (PMMA): 0.35 ± 0.07	Polishing + Surface Sealant (C) Duo Cad (PMMA): 0.32 ± 0.06	Polishing (D) Temporis (Hybrid composite): 0.23 ± 0.04	Polishing + Surface Sealant (D) Temporis (Hybrid composite): 0.23 ± 0.03	Polishing and surface sealant	N/A	Contact profilometer (MarSurf PS10; Mahr GmbH)	Ra after polishing only: Conventional PMMA > CAD/CAM Milled PMMA > Conventional Bisacrylic > 3D-Printed hybrid composite Significant reduction in SR after application of surface sealant for all groups except in 3D-printed materials.
Atria et al., 2020 [42]	Ra before: (A) Marche (1.3 mm): 0.22 ± 0.01 Marche (0.6 mm): 0.26 ± 0.02 (B) Protemp (1.3 mm): 0.18 ± 0.01 Portemp (0.6 mm): 0.20 ± 0.02 Ra after Thermocycling: (A) Marche (1.3 mm): 0.31 ± 0.02 Marche (0.6 mm): 0.31 ± 0.02 (B) Protemp (1.3 mm): 0.23 ± 0.01 Portemp (0.6 mm): 0.25 ± 0.02 Δ Ra (A) Marche (1.3 mm): 0.09 ± 0.02 Marche (0.6 mm): 0.05 ± 0.02 (B) Protemp (1.3 mm): 0.05 ± 0.02 Portemp (0.6 mm): 0.04 ± 0.02		Ra before: (C) TelioCAD (1.3 mm): 0.20 ± 0.02 TelioCAD (0.6 mm): 0.20 ± 0.02 Ra after Thermocycling: (C) TelioCAD (1.3 mm): 0.19 ± 0.01 TelioCAD (0.6 mm): 0.20 ± 0.01 Δ Ra (C) TelioCAD (1.3 mm): −0.01 ± 0.02 TelioCAD (0.6 mm): 0.00 ± 0.01		Ra before: (C) Raydent (1.3 mm): 0.26 ± 0.03 Raydent (0.6 mm): 0.21 ± 0.02 Ra after Thermocycling: (C) Raydent (1.3 mm): 0.54 ± 0.03 Raydent (0.6 mm): 0.60 ± 0.03 Δ Ra (C) Raydent (1.3 mm): 0.28 ± 0.02 Raydent (0.6 mm): 0.38 ± 0.03		Polishing	Thermocycling: 6000 cycles at 5–55 °C	Rugosimeter (SRT 1200; PCE instruments)	Δ Ra: 3D-Printed hybrid composite > Conventional PMMA > Conventional Bis-acryl resin > CAD/CAM PMMA.
Myagmar et al., 2021 [47]	Ra Before Wear test 0.26 ± 0.02	After wear test (A) 30,000 cycles: 0.92 ± 0.09 (B) 60,000 cycles: 1.63 ± 0.44	Before Wear test 0.19 ± 0.03	After wear test (A) 30,000 cycles: 0.88 ± 0.05 (B) 60,000 cycles: 1.27 ± 0.49	Before Wear test 0.13 ± 0.01	After wear test (A) 30,000 cycles: 0.48 ± 0.06 (B) 60,000 cycles: 0.58 ± 0.06	Polishing	Simulated chewing subjected to 30,000 or 60,000 cycles of chewing simulation against the metal abrader	Confocal laser scanning microscope (LSM 800 MAT, Zeiss)	Ra after wearing: Conventional PMMA > CAD/CAM Milled PMMA > 3D-Printed PMMA

**Table 10:** Wear Resistance Results.

Author and Year	Mean/Medians and Interquartile Ranges (IQRs) of the Volume Loss (mm^3^) for Conventional	Mean/Medians and Interquartile Ranges (IQRs) of the Volume Loss (mm^3^) for CAD/CAM Milled	Mean/Medians and Interquartile Ranges (IQRs) of the Volume Loss (mm^3^) for 3D-Printed	Mean/Medians and IQRs of the Maximal Depth Loss (mm) for Conventional	Mean/Medians and IQRs of the Maximal Depth Loss (mm) for CAD/CAM Milled	Mean/Medians and IQRs of the Maximal Depth Loss (mm) for 3D-Printed	Parameters of the Chewing Simulator	Measuring Device	Authors Suggestions/Conclusions
Park et al., 2018 [26]	Median and IQR Jet (PMMA) Against Zirconia abrader: 1.06 (0.93–1.63) Against metal abrader: 1.06 (0.73–2.30)	Median and IQR Vipiblock (PMMA) Against Zirconia abrader: 1.20 (0.90–1.42) Against metal abrader: 1.11 (0.63–1.81)	Median and IQR C&B (PMMA) Against Zirconia abrader: 1.11 (0.96–1.50) Against metal abrader: 1.22 (0.47–2.20)	Median and IQR Jet (PMMA) Against Zirconia abrader: 0.35 (0.32–0.41) Against metal abrader: 0.38 (0.25–0.57)	Median and IQR Vipiblock (PMMA) Against Zirconia abrader: 0.35 (0.30–0.41) Against metal abrader: 0.38 (0.28–0.51)	Median & IQR C&B (PMMA) Against Zirconia abrader: 0.36 (0.32–0.43) Against metal abrader: 0.42 (0.22–0.56)	chewing simulator CS-4.8, SD Vertical load: 5 Kg (49 N) lateral movement: 2 mm Abrasion cycles: 30,000	3-axis blue LED light scanner (Identica Hybrid)	Wear resistance of the 3D-printed PMMA resin material is comparable to CAD/CAM milled PMMA or the conventionally fabricated PMMA resin materials. 3D-printed resins provide adequate wear resistance for dental use.
Mayer et al., 2020 [50]	N/A	Mean ± SD Against metal abrader: (A) Telio CAD −0.421 ± 0.216	Mean ± SD Against metal abrader: (B) Freeprint temp CEN: −0.168 ± 0.078 ISO: −0.137 ± 0.064 YEL: −0.134 ± 0.052 (C) GC Temp PRINT CEN: −0.193 ± 0.075 ISO: −0.283 ± 0.13 YEL: −0.236 ± 0.037 (D) Next dent C&B MFH CEN: −0.246 ± 0.072 ISO: −0.142 ± 0.028 YEL: −0.15 ± 0.065	N/A	Mean ± SD Against metal abrader: (A) Telio CAD disc −0.181 ± 0.071	Mean ± SD Against metal abrader: (B) Freeprint temp CEN: −0.115 ± 0.026 ISO: −0.100 ± 0.024 YEL: −0.107 ± 0.023 (C) GC Temp PRINT CEN: −0.145 ± 0.027 ISO: −0.147 ± 0.034 YEL: −0.154 ± 0.032 (D) Next dent C&B MFH CEN: −0.148 ± 0.025 ISO: −0.104 ± 0.027 YEL: −0.131 ± 0.031	Chewing simulator CS-4, SD vertical load: 50 N lateral movement: 0.7 mm masticatory cycles: 480,000 Simultaneous thermocycling in distilled water between 10° and 55 °C with a duration of 60 s for each cycle	laser scanner (LAS-20; SD)	Two body Wear resistance: 3D-Printed PMMA > CAD/CAM Milled PMMA No significant effect of cleaning method on wear resistance of 3D-printed materials.
Myagmar et al., 2021 [47]	Mean ± SD (A) JetTM After 30,000 cycles: 0.11 ± 0.01 After 60,000 cycles: 0.44 ± 0.01	Mean ± SD (B) Yamahachi PMMA After 30,000 cycles: 0.06 ± 0.01 After 60,000 cycles: 0.21 ± 0.02	Mean ± SD (C) NextDent C&B After 30,000 cycles: 0.08 ± 0.09 After 60,000 cycles: 0.10 ± 0.01	N/A	N/A	N/A	chewing simulator CS-4.8, SD vertical load of 5 kg 5-mm vertical descending movement 2 mm horizontal movement Simultaneous thermocycling in distilled water between 5° and 55 °C Two subgroups abraded for: 30,000 or 60,000 cycles	multiline blue LED light scanner (D1000, 3Shape)	wear resistance: 3D-Printed PMMA > CAD/CAM milled PMMA > conventional PMMA
Kessler et al., 2019 [27]	N/A	N/A	N/A	Mean Wear loss in μm (A) TetricEvoCeram: Average Mean Wear loss: 50 ± 15 μm Mean Wear loss (i) 50,000 cycles: 13 ± 5 (ii) 100,000 cycles: 23 ± 2.3 (iii) 150,000 cycles: 35 ± 9 (iv) 200,000 cycles: 50 ± 15	Mean Wear loss in μm (B) Telio CAD Average Mean Wear loss: <236 ± 31 μm Mean Wear loss (i) 50,000 cycles: 56 ± 5 (ii) 100,000 cycles: 111 ± 210 (iii) 150,000 cycles: 178 ± 10 (iv) 200,000 cycles: 236 ± 31	Mean Wear loss in μm (C) 3Delta temp Average Mean Wear loss: <62 ± 4 μm Mean Wear loss: (i) 50,000 cycles: 16 ± 2 (ii) 100,000 cycles: 32 ± 1.4 (iii) 150,000 cycles: 48 ± 3 (iv) 200,000 cycles: 62 ± 4 (D) Nextdent C&B Average Mean Wear loss: < 255 ± 13 μm Mean Wear loss: (i) 50,000 cycles: 66 ± 5 (ii) 100,000 cycles: 134 ± 4.6 (iii) 150,000 cycles: 200 ± 7 (iv) 200,000 cycles: 255 ± 13 (E) Freeprint temp Average Mean Wear loss:< 257 ± 24 μm Mean Wear loss (i) 50,000 cycles: 57 ± 5 (ii) 100,000 cycles: 125 ± 2.8 (iii) 150,000 cycles: 191 ± 6 (iv) 200,000 cycles: 257 ± 24	Antagonist wheel rotated 15% slower than the sample wheel and pressed against it with a spring force of 15 N.	LaserScan3D, Willytec	The average mean wear: 3D-printed Freeprint temp> 3D-Printed NextDent > CAD/Cam Milled TelioCAD > 3D-printed 3Delta temp > conventional TetricEvoCeram Wear resistance of 3D-printed comparable to others. Addition of filler increases wear resistance. So, materials with high filler content are recommended for fabricating long-term provisional restorations.

N/A: Not Applicable.

**Table 11:** Flexural strength (FS) results.

Author and Year	Mean/Median of Maximum Force at Fracture for Conventional Resin	Mean/Median of Maximum Force at Fracture for CAD/CAM Milled Resin	Mean/Median of Maximum for 3D-Printed Resin	Exposure Agent/Aging Technique	Testing Machine Used	Authors Suggestions/Conclusions
Park et al., 2020 [43]	Medians and IQRs of FS: (A) Jet Tooth ShadeTM Powder: 543 N [IQR: 429–701]	Medians and IQRs of FS: (B) ViPi: 1232 N [IQR: 1193–1258]	Medians and IQRs of FS: (C) NextDent C&B: 1189 N [IQR: 1110–1283] (D) Standard (GPGR04): 1323 N [IQR: 1245–1377] (E) PLA: Data N/A	N/M	Universal testing machine	FS: 3D-printed PPMA ((D) > CAD/CAM milled PMMA > 3D-Printed PMMA ((C) > conventional PMMA The (FDM) group 3D-printed Polylactic-acid-based restoration did not fracture but was dented
Crenn et al., 2022 [29]	Mean FS: (A) Integrity: 115.4 ± 20.5 MPa (B) Unifast: 85.79 ± 6.00 MPa	N/A	Mean FS: (C) PLA: 115.8 ± 2.11 MPa (D) Temporary CB: 134.9 ± 17.51 MPa	N/M	Universal testing machine	FS: 3D-Printed SLA Polymer > 3D-Printed PLA ≥ Conventional Bis-acrylic > conventional MMA
Tas¸ın et al., 2022 [30]	Median in MPa (A) Temdent Classic Thermocycling: (i) 0 cycles: 68 (ii) 2500 cycles: 62 (iii) 10,000 cycles: 49 (B) Protemp: Thermocycling: (i) 0 cycles: 113 (ii) 2500 cycles: 108 (iii) 10,000 cycles: 99	Median in MPa (C) Duo Cad: Thermocycling: (i) 0 cycles: 127 (ii) 2500 cycles: 122 (iii) 10,000 cycles: 117	Median in MPa (D) Temporis: Thermocycling: (i) 0 cycles: 125 (ii) 2500 cycles: 125 (iii) 10,000 cycles:116	Thermocycling control (0 cycles), 2500 cycles, and 10,000 cycles	Universal testing machine	FS at all thermocycling periods: CAD/CAM milled PMMA ≈ 3D-Printed composite > conventional bis-acrylic > conventional MMA Thermocycling periods influence the flexural strength of each tested group
Digholkar et al., 2016 [36]	Mean FS: (A) Heat-activated PMMA: 95.58 ± 12.444 MPa	Mean FS: (B) Ceramill TEMP: 104.20 ±12.777 MPa	Mean FS: (C) E-Dent 100: 79.54 ± 10.130 MPa	N/M	Universal testing machine	FS: CAD/CAM-milled PMMA > Conventional heat activated PMMA > 3D-printed Microhybrid filled composite
Simoneti et al., 2022 [53]	Mean FS in MPa: (A) Dencor (PMMA): 69.2 ± 8.8 (B) Yprov Bis-acryl (Bis-acryl resin): 75.0 ± 8.2	N/A	Mean FS in MPa: (C) PA2201 (SLS resin): 77.3 ± 3.1 (D) Gray Resin (SLA resin): 48.9 ± 1.2	Mechanical fatigue simulation: 120,000 cycles performed to simulate 6 months of clinical use	Universal testing machine	FS: 3D-Printed SLS > conventional Bis-acrylic > conventional PMMA > 3D-Printed SLA resin
Pantea et al., 2022 [31]	Mean FS in MPa: (A) Duracyl: 88 ±10 (B) Superpont C+B: 76 ± 7	N/A	Mean FS in MPa: (C) NextDent C&B MFH: 141 ± 17 (D) HARZ Labs Dental Sand: 143 ± 15	N/M	Universal testing machine	Flexural strength: 3D-Printed PMMA > conventional PMMA

IQR: Interquartile range; N/A: Not Applicable; N/M: Not Mentioned.

**Table 12:** Elastic Modulus Results.

Author and Year	Mean Elastic Modulus of Conventional Resin (Mpa)	Mean Elastic Modulus for CAD/CAM Milled Resin (MPa)	Mean Elastic Modulus for 3D-Printed Resin (MPa)	Exposure Agent/Aging Technique	Testing Machine Used	Authors Suggestions/Conclusions
Tahayeri et al., 2018 [37]	(A) Jet ~1500 (B) Integrity ~2700	N/A	(C) NextDent C&B resin ~1700	N/M	Universal testing machine	Elastic Modulus: Conventionally fabricated bis-acrylic > 3D-printed PMMA > conventionally fabricated PMMA
Simoneti et al., 2022 [53]	(A) Decor Acrylic resin: 859.4 ± 46.3 (B) Yprov Bisacryl: 997.3 ±108.5	N/A	(C) PA2201 (SLS resin): 452.4 ± 35.8 (D) Gray Resin (SAL resin): 513.3 ± 29.7	Mechanical fatigue simulation: 120,000 cycles, Simulating 6 months of clinical use	Universal testing machine	Elastic Modulus: Conventionally fabricated PMMA and bis-acrylic > 3D-printed PMMA
Crenn et al., 2022 [29]	(A) Integrity: 3977 ± 878.2 (B) Unifast: 2382 ± 225.8	N/A	(C) PLA Bio source: 3784 ± 98.9 (D) Temporary CB: 4607 ± 213.8	Storage at ambient temperature for 1 week	Universal testing machine	Elastic Modulus: 3D-printed esters > Conventional bis-acrylic > 3D-Printed poly lactic > Conventional MMA.

N/A: Not Available; N/M: Not Mentioned.

**Table 13:** Toughness Results (MJ/m^3^).

Author and Year	Toughness for Conventional Resin	Toughness for CAD/CAM Milled Resin	Toughness for 3D-Printed Resin	Exposure Agent/Aging Technique	Testing Machine Used	Authors Suggestions/Conclusions
Tas¸ın et al., 2022 [30]	Median in MJ/m^3^ (A) Temdent Classic (PMMA) Thermocycling: (i) 0 cycles: 1.82 (ii) 2500 cycles: 1.31 (iii) 10,000 cycles: 0.96 (B) Protemp (Bis-Acryl) Thermocycling: (i) 0 cycles: 2.47 (ii) 2500 cycles: 2.47 (iii) 10,000 cycles: 1.54	Median in MJ/m^3^ (C) Duo Cad (PMMA): Thermocycling: (i) 0 cycles: 4.93 (ii) 2500 cycles: 4.59 (iii) 10,000 cycles: 3.70	Median in MJ/m^3^ (D) Temporis (composite resin): Thermocycling: (i) 0 cycles: 3.63 (ii) 2500 cycles: 3.09 (iii) 10,000 cycles: 2.20	Thermocycling	Universal testing machine	Toughness after thermocycling 10,000 cycles: CAD/CAM Milled PMMA > 3D-printed composite resin > conventional Bis-acrylic > conventional PMMA

**Table 14:** Peak Stress Results.

Author and Year	Mean Peak Stress for Conventional Resin	Mean Peak Stress for CAD/CAM Milled Resin	Mean Peak Stress for 3D-printed Resin	Exposure Agent/Aging Technique	Testing Machine Used	Authors Suggestions/Conclusions
Tahayeri et al., 2018 [37]	(A) Jet: ≅65 MPa (B) Integrity: ≅90 MPa	N/A	(C) NextDent C&B resin: ≅95 MPa	N/M	Universal testing machine	Peak stress: 3D-printed NextDent > Conventionally fabricated Integrity > conventionally fabricated Jet
Simoneti et al., 2022 [53]	(A) Dencor (PMMA): 114.6 ± 14.6 N (B) Yprov Bisacryl (Bis-acryl resin) 131.1 ± 2.2 N	N/A	(C) PA 2201 (SLS resin): 133.7 ± 4.4 N (D) Gray Resin (SLA resin): 58.7 ± 2.2 N	Mechanical fatigue simulation: 120,000 cycles simulating 6 months of clinical use	Universal testing machine	Peak stress: 3D-Printed SLS > Conventional Bisacrylic > conventional PMMA > 3D-Printed SLA

N/A: Not Applicable; N/M: Not Mentioned.

**Table 15:** Resilience Results (MJ/m^3^).

Author and Year	Resilience for Conventional Resin	Resilience for CAD/CAM Milled Resin	Resilience for 3D-Printed Resin	Exposure Agent/Aging Technique	Testing Machine Used	Authors Suggestions/Conclusions
Tas¸ın et al., 2022 [30]	Median in (MJ/m^3^) (A) Temdent Classic (PMMA): Thermocycling: (i) 0 cycles: 0.77 (ii) 2500 cycles: 0.64 (iii) 10,000 cycles: 0.53 (B) Protemp (Bis-Acryl): Thermocycling: (i) 0 cycles: 0.98 (ii) 2500 cycles: 0.81 (iii) 10,000 cycles: 0.72	Median in (MJ/m^3^) (C) Duo Cad (PMMA): Thermocycling: (i) 0 cycles: 1.04 (ii) 2500 cycles: 0.93 (iii) 10,000 cycles: 0.85	Median in (MJ/m^3^) (D) Temporis (composite resin): Thermocycling: (i) 0 cycles: 1.12 (ii) 2500 cycles: 1.03 (iii) 10,000 cycles: 0.74	Thermocycling	Universal testing machine	Resilience results after thermocycling for 10,000 cycles: CAD/CAM milled PMMA > 3D-Printed composite resin > Conventional Bisacrylic > conventional PMMA

## 3. Results

### 3.1. Identification and Screening

This literature review compared the physical and mechanical properties of resins used for fabricating provisional crowns and FDPs by 3D-printing with those provisional resins used for CAD/CAM milling and other conventional techniques. For ease of understanding, the results of each physical and mechanical property were tabulated in separate tables (Table 5, Table 6, Table 7, Table 8, Table 9, Table 10, Table 11, Table 12, Table 13, Table 14 and Table 15).

**Figure 1:**
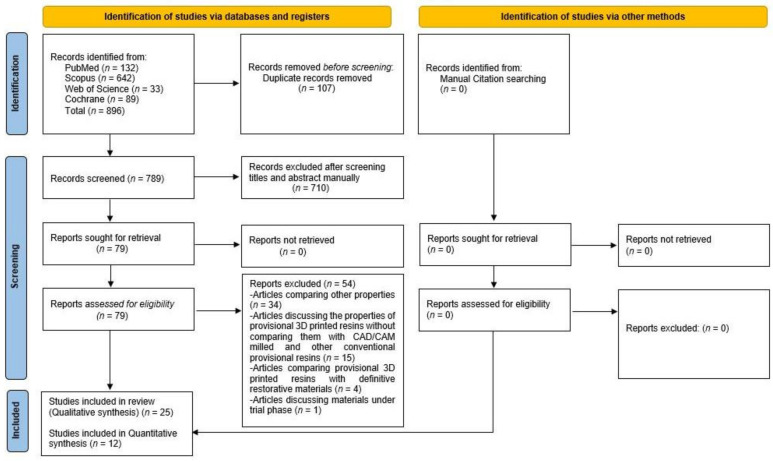
Article selection strategy based on PRISMA guidelines.

Eight hundred and ninety-six titles were recognized from the primary search on the selected electronic databases. On checking, 107 titles were found to be duplicates and were excluded. After reviewing the titles and abstracts, 710 articles were rejected as they did not meet the inclusion and exclusion criteria. Full texts of the remaining 79 articles were reviewed, and secondary articles were searched manually from the references of these articles, but no more relevant articles were found. Out of the selected 79 articles, 15 were rejected, as they were discussing the properties of provisional 3D-printed resins without comparing them with CAD/CAM milled and other conventional provisional resins. Thirty-four articles were rejected as they compared other properties (other than physical and mechanical), and four were rejected as they were comparing provisional 3D-printed resins with definitive restorative materials. Finally, one article was rejected as it discussed the properties of 3D-printed resins under the trial phase. Thus, 25 articles were finally included in this systematic review for qualitative analysis. Out of 25 articles, only 12 provided comparative data and were included for quantitative analysis (Figure 1).

### 3.2. Quality Assessment of Included Studies

All twenty-five studies included in this review were in vitro studies. A total of 221 out of 375 (58.93%) entries were positively reported. All studies reported items related to abstract, introduction, intervention, outcome, statistical method, and results (Items 1–4, 10, and 11). Fifteen studies addressed the trial limitations (Item 12) and provided information related to funding sources (Item 13). Only six studies mentioned the procedure of calculating the sample size of the specimens (Item 5), while five studies gave details related to the accessibility of the full trial protocol (Item 14). Only four studies described the method used to generate random allocation sequence (Item 6), with one of them reporting the allocation concealment mechanism briefly (Item 7). Details related to the blinding of the examiners and the details of the researcher who generated the random allocation were not reported by any of the studies (Item 8 and 9) (Table 4).

### 3.3. Study Characteristics

The majority of the studies (21 out of 25) included in this review were published between 2020 and 2022, while four were published between the years 2016 and 2019. All the included articles were in vitro studies. Nineteen articles analyzed and compared the mechanical properties, four analyzed physical properties, and two articles analyzed both physical and mechanical properties. Some of the studies focused on one particular character, while others studied multiple characteristics at the same time (Table 3).

### 3.4. Results of Studies Analyzing the Physical Properties

#### 3.4.1. Color Change

Five studies compared the change in the color values of 3D-printed interim resins with other materials (Table 5).

(i) Comparing the change in color values of MMA-based 3D-printed provisional resins: Three studies reported a greater change in the color values of MMA-based 3D-printed resins when compared to CAD/CAM milled PMMA resins [28,44,45].

**Figure 2:**
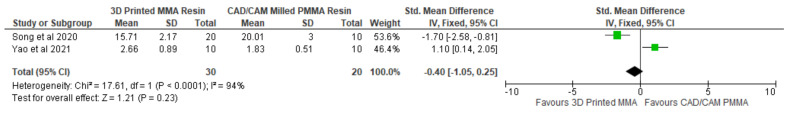
Forest plot comparing color change between 3D-Printed MMA Resin and CAD/CAM milled PMMA resin.

Two studies provided data for the meta-analysis to compare color changes between 3D-Printed MMA Resins and CAD/CAM Milled PMMAs. There was a statistically significant heterogeneity between the studies, with I^2^ = 94%. The results were inconclusive, favoring 3D-Printed MMA (*p* = 0.23) (Figure 2).

(ii) Comparing the change in color values of hybrid composite-based 3D-printed provisional resins: Studies by Atria et al. [42] reported a greater change in color for hybrid composite-based 3D-printed provisional resins when compared to conventional bis-acrylic and PMMA resins. On the contrary, Taşın et al. [48] and Song et al. [44] reported greater change in color for conventional resins. Compared to CAD/CAM milled PMMA resins, a greater change in color was reported in 3D-printed hybrid composite resins [15,19].

**Figure 3:**
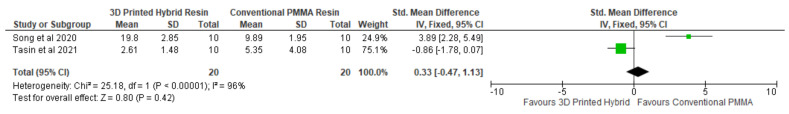
Forest plot comparing color change between 3D-printed hybrid resin and conventional PMMA resin.

Two studies provided data for the meta-analysis to compare color changes between 3D-printed hybrid resin and conventional PMMA resin. There was a statistically significant heterogeneity between the studies, with I^2^ = 96%. The results were inconclusive, favoring conventional PMMA resin (*p* = 0.40) (Figure 3).

**Figure 4:**
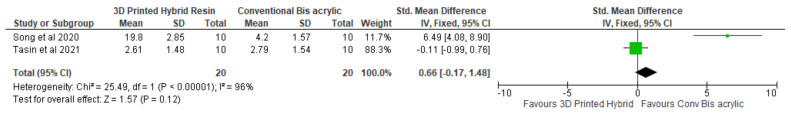
Forest plot comparing color change between 3D-printed hybrid resin and conventional PMMA resin.

Two studies provided data for meta-analysis to compare color changes between 3D-printed hybrid resin and conventional bBis-acrylic resin. There was a statistically significant heterogeneity between the studies, with I^2^ = 96%. The results were inconclusive, favoring conventional bBis-acrylic resin (*p* = 0.12) (Figure 4).

#### 3.4.2. Water Sorption and Solubility

Two studies compared the water sorption and solubility of 3D-printed interim resins with other materials (Table 6). The water sorption of 3D-printed PMMA resins was reported to be higher than conventional polycarbonate resins and lower than conventional PMMA resins [28]. For 3D-printed photopolymer resins, the water sorption was reported to be higher than conventional bis-acrylic and CAD/CAM milled PMMA resins and lower than conventional PMMA resins [44]. The solubility of the 3D-printed PMMA resins was reported to be higher than conventional polycarbonate and PMMA resins [28]. For 3D-printed photopolymer resins, the solubility was higher than conventional PMMA, conventional bis-acrylic, and CAD/CAM milled PMMA resins [44].

### 3.5. Results of Studies Analyzing the Mechanical Properties

#### 3.5.1. Fracture Strength

Eight studies analyzed and compared the fracture strength of 3D-printed resins with CAD/CAM milled and/or conventionally fabricated resins used for the fabrication of provisional crowns and FDPs (Table 7).

(i) Comparing the fracture strength of PMMA-based 3D-printed provisional resins: Three studies reported higher FS when compared to PMMA-based CAD/CAM milled resins [38,39,40]. One study reported contrasting results of lower FS when compared to PMMA-based CAD/CAM milled resins [50], and one study each reported higher FS when compared to conventional MMA [39] and bis-acrylic resins [40]. A study by Reeponmaha et al. [16] reported higher FS MMA-based 3D-printed resins when compared to PMMA-based CAD/CAM milled and conventional resins.

**Figure 5:**
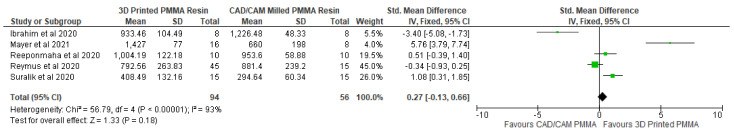
Forest plot comparing fracture strength between 3D-printed PMMA resin and CAD/CAM milled PMMA resin.

Five studies provided data for the meta-analysis to compare the fracture strength between 3D-printed PMMA resin and CAD/CAM milled PMMA resin. There was a statistically significant heterogeneity between the studies, with I^2^ = 93%. The results were inconclusive, favoring 3D-printed PMMA (*p* = 0.18) (Figure 5).

**Figure 6:**
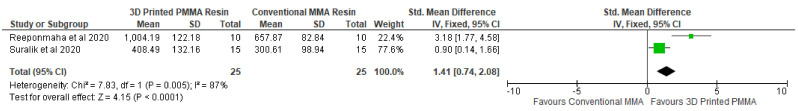
Forest plot comparing fracture strength between 3D-printed PMMA resin and conventional PMMA resin.

Two studies provided data for the meta-analysis to compare the fracture strength between 3D-Printed PMMA resin and conventional PMMA resin. There was a statistically significant heterogeneity between the studies, with I^2^ = 87%. However, both the studies favored 3D-printed PMMA resin, and the 95% confidence interval did not include 0, i.e., no effect. Thus, the pooled estimate favored 3D-printed PMMA resin with *p* < 0.0001 (Figure 6).

(ii) Comparing the fracture strength of bis-acrylic and other photopolymer hybrid 3D-printed provisional resins: the FSs of 3D-printed bis-acrylic resin [48], micro-hybrid resin [46], photopolymer resin [52], and UDMA-based resins [50] were reported to be lower than PMMA-based CDA/CAM resins. A study by Henderson et al. [51] reported that bis-acrylic-based 3D-printed resins have lower FS when compared to bis-acrylic-based conventional resins.

**Figure 7:**
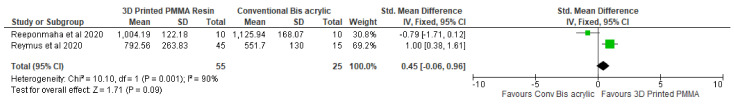
Forest plot comparing fracture strength between 3D-printed PMMA resin and conventional bBis-acrylic resin.

Two studies provided data for the meta-analysis to compare the fracture strength between 3D-printed PMMA resin and conventional bis-acrylic resin. There was a statistically significant heterogeneity between the studies, with I^2^ = 90%. The results were inconclusive, favoring 3D-printed PMMA resin (*p* = 0.09) (Figure 7).

#### 3.5.2. Microhardness

Four studies compared the microhardness of 3D-printed interim resins with other materials. Two studies measured Vickers hardness [29,53], while the other two measured knop hardness [36,49] (Table 8).

(i) Comparing the hardness of MMA-based 3D-printed provisional resins: Two studies reported lower hardness values of MMA-based 3D-printed resins when compared to conventional MMA [49,53] and conventional bis-acrylic interim resins [53], respectively. Moreover, a study by Revilla-León et al. [49] reported higher hardness values for 3D-printed MMA-based interim resins when compared to conventional bis-acrylic interim resins.
(ii) Comparing hardness of micro-filled and polylactic-acid-based 3D-printed provisional resins: Digholkar et al. [36] reported higher hardness values for 3D-printed micro-filled resins when compared to conventional PMMA-based interim resins, whereas Crenn et al. [29] reported PMMA-based conventional resins to have higher hardness values when compared to 3D-printed polylactic-acid-based interim resins.

#### 3.5.3. Surface Roughness

Four studies compared the surface roughness of 3D-printed interim resins with other materials (Table 9).

(i) Comparing the surface roughness of MMA-based 3D-printed provisional resins: Myagmar et al. [47] reported lower surface roughness values for MMA-based 3D-printed resins compared to PMMA-based conventional resins and CAD/CAM milled interim resins.

**Figure 8:**
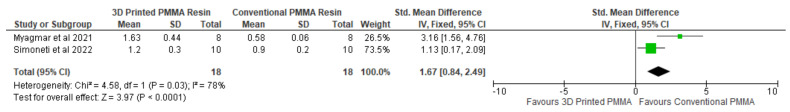
Forest plot comparing surface roughness between 3D-printed PMMA resin and conventional PMMA resin.

Two studies provided data for meta-analysis to compare Surface Roughness between 3D-printed PMMA resin and conventional PMMA resin. There was a statistically significant heterogeneity between the studies, with I^2^ = 78%. Both studies favored the conventional PMMA with a 95% confidence interval. The pooled estimate favored conventional PMMA resin with a *p*-value < 0.0001 (Figure 8).

**Figure 9:**
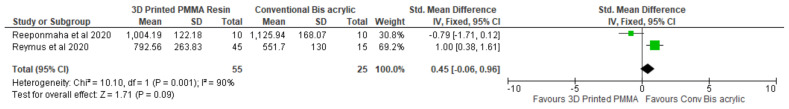
Forest plot comparing surface roughness between 3D-printed PMMA resin and conventional bBis-acrylic resin.

Two studies provided data for the meta-analysis to compare the surface roughness between 3D-printed PMMA resin and conventional bis-acrylic resin. There was a statistically significant heterogeneity between the studies, with I^2^ = 90%. The results were inconclusive, favoring conventional bis-acrylic resin (*p* = 0.09) (Figure 9).

(ii) Comparing the surface roughness of hybrid and other 3D-printed provisional resins: One study [42] showed that hybrid 3D-printed resins have a higher surface roughness when compared to conventional PMMA, conventional bis-acrylic, and CAD/CAM milled PMMA-based resins. However, the results of a study by Taşın et al. [48] gave contradictory results, with hybrid 3D-printed resins displaying a lower surface roughness when compared to conventional PMMA, conventional bis-acrylic, and CAD/CAM milled PMMA-based resins. Simoneti et al. [53] reported that the surface roughness of SLS 3D-printed resins was higher, and that of SLA-based 3D-printed resins was lower when compared to conventional PMMA and bis-acrylic-based interim resins.

**Figure 10:**
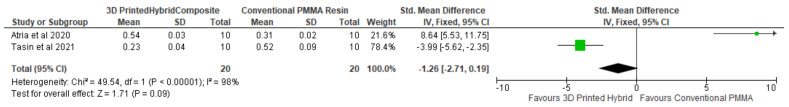
Forest plot comparing the surface roughness between 3D-printed hybrid composite resin and conventional PMMA resin.

Two studies provided data for the meta-analysis to compare the surface roughness between 3D-printed hybrid composite resins and conventional PMMA resins. There was a statistically significant heterogeneity between the studies, with I^2^ = 98%. The results were inconclusive, favoring 3D-printed hybrid composite resin (*p* = 0.09) (Figure 10).

**Figure 11:**
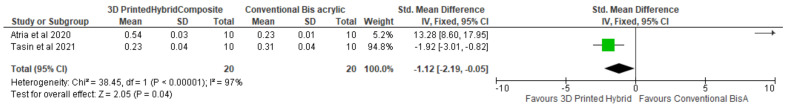
Forest plot comparing surface roughness between 3D-hybrid composite resin and conventional bBis-acrylic resin.

Two studies provided data for the meta-analysis to compare the surface roughness between 3D-hybrid composite resin and conventional bis-acrylic resin. There was a statistically significant heterogeneity between the studies, with I^2^ = 97%. The studies showed varied results, one favoring each side. The pooled estimate favored 3D-printed hybrid composite resin with a *p*-value = 0.04 (Figure 11).

**Figure 12:**
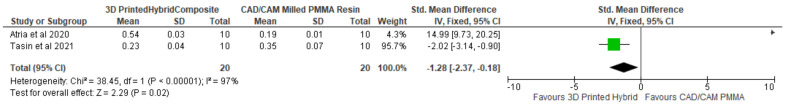
Forest plot comparing surface roughness between 3D-hybrid composite resin and CAD/CAM milled PMMA resin.

Two studies provided data for the meta-analysis to compare the surface roughness between 3D-hybrid composite resin and CAD/CAM milled PMMA resin. There was a statistically significant heterogeneity between the studies, with I^2^ = 97%. The studies showed varied results, one favoring each side. The pooled estimate favored 3D-printed hybrid composite resin with a *p*-value = 0.02 (Figure 12).

#### 3.5.4. Wear Resistance

Four studies compared the wear resistance of 3D-printed interim resins with other materials (Table 10).

Comparing the wear resistance of MMA-based 3D-printed provisional resins: The wear resistance of MMA-based 3D-printed provisional resins was reported to be higher than the wear resistance of PMMA-based conventional and CAD/CAM milled 3D-printed interim resins [26,27,47,50].

**Figure 13:**
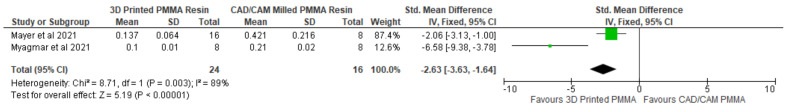
Forest plot comparing wear resistance between 3D-printed PMMA resin and CAD/CAM milled PMMA resin.

Two studies provided data for the meta-analysis to compare the wear resistance between 3D-printed PMMA resin and CAD/CAM milled PMMA resin. There was a statistically significant heterogeneity between the studies, with I^2^ = 89%. Both studies favored the 3D-printed PMMA resin with a 95% confidence interval. The pooled estimate favored 3D-printed PMMA resin with a *p*-value < 0.00001 (Figure 13).

#### 3.5.5. Flexural Strength

Six studies compared the flexural strength values of 3D-printed interim resins with other materials (Table 11).

(i) Comparing the flexural strength of MMA-based 3D-printed provisional resins: Two studies reported higher flexural strength values of MMA-based 3D-printed resins when compared to conventional MMA [31,43] and CAD/CAM milled PMMA resin [43].

**Figure 14:**
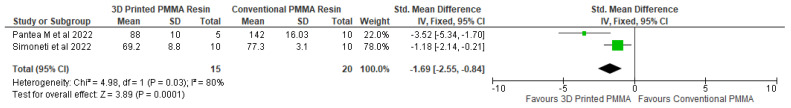
Forest plot comparing flexural strength between 3D-printed PMMA resin and CAD/CAM milled PMMA resin.

Two studies provided data for the meta-analysis to compare the flexural strength between 3D-Printed PMMA resin and CAD/CAM milled PMMA resin. There was a statistically significant heterogeneity between the studies, with I^2^ = 80%. Both studies favored the 3D-printed PMMA resin with a 95% confidence interval. The pooled estimate favored 3D-printed PMMA resin with a *p*-value < 0.0001 (Figure 14).

(ii) Comparing the flexural strength of composite-based 3D-printed provisional resins: Taşın et al. reported higher flexural strength values of composite-based 3D-printed resins compared to conventional MMA and conventional bis-acrylic-based resins [30], whereas a study by Digholkar et al. reported lower flexural strength values compared to CAD/CAM milled PMMA and conventional heat cure PMMA-based resins [37]. Contrasting results were reported when the flexural strengths of SLA 3D-printed resins were compared with conventional PMMA and bis-acrylic resins. Crenn et al. [29] reported higher flexural strength values for 3D-printed resins, while Simoneti et al. [53] reported higher values for conventional resins.

#### 3.5.6. Elastic Modulus

Five studies compared the elastic modulus of 3D-printed interim resins with other materials (Table 12).

(i) Comparing the elastic modulus of MMA-based 3D-printed provisional resins: Two studies reported higher elastic modulus values of MMA-based 3D-printed resins when compared to conventional MMA [31,37], whereas a study by Simoneti et al. reported lower elastic modulus values when compared to conventional PMMA-based resins [53]. Two studies reported lower elastic modulus values of MMA-based 3D-printed resins compared to conventional bis-acrylic resins [37,53].
(ii) Comparing the elastic modulus of composite-based, ester-based, and polylactic-acid-based 3D-printed provisional resins: Crenn et al. [29] reported higher elastic modulus values for ester-based and polylactic-acid-based 3D-printed resins when compared to conventional PMMA and bis-acrylic-based resins. Taşın et al. [30] reported higher elastic modulus values for composite-based 3D-printed resins compared to conventional PMMA, CAD/CAM PMMA, and conventional bis-acrylic-based resins.

#### 3.5.7. Toughness, Peak Strain, and Resilience

Two studies compared the peak strain values, and one each studied toughness and resilience of 3D-printed interim resins with other materials (Table 13, Table 14 and Table 15).

Taşın et al. [30] reported that the resilience and toughness of 3D-printed composite resins is higher than conventional PMMA and bis-acrylic resins but lower than CAD/CAM milled PMMA resins.

When peak stress values were compared, Tahayeri et al. [37] reported higher values for 3D-printed PMMA when compared to conventional bis-acrylic and PMMA-based resins. The study by Simoneti et al. [53] reported that peak stress values for conventional resins (bis-acrylic and PMMA) were higher than 3D-printed SLA resins but lower than 3D-printed SLS resins.

## 4. Discussion

The introduction of CAD/CAM technology in the field of fixed prosthodontics has improved the quality of treatment provided to the patients [54]. This systematic review and meta-analysis is the first of its kind to analyze and document all the available studies comparing the mechanical and/or physical properties of the 3D-printed provisional crown and FPD materials with CAD/CAM milled and/or conventional provisional resins. All twenty-five papers included were in vitro studies [16,26,27,28,29,30,31,35,36,37,38,39,40,41,42,43,44,45,46,47,48,49,50,51,52]. The overall findings reveal that the mechanical and physical properties of the provisional crown and FDP materials are affected by the technique of fabrication and composition of the tested materials. Three-dimensionally printed provisional materials have shown significantly different mechanical and physical properties. Thus, the tested null hypothesis is rejected. The mechanical and physical properties of 3D-printed provisional resins in comparison to conventional and CAD/CAM milled will be discussed.

### 4.1. Physical Properties

Three physical properties (color stability, solubility, and water sorption) were evaluated in the selected articles. In general, most of the studies reported that, irrespective of the composition, the 3D-printed provisional crown and FDP materials displayed poor physical properties when compared to CAD/CAM milled and conventionally processed provisional restorative materials. Three studies [28,44,45] that compared the color stability of 3D-printed PMMA resins reported that they have poor color stability when compared to CAD/CAM milled PMMA resins. The studies by Atria et al. [15] and Taşın et al. [19] reported a poor color stability of 3D-printed hybrid composite resins compared to CAD/CAM milled PMMA, conventional PMMA, and conventional bis-acrylic provisional resins. However, two studies [44,48] reported better color stability for 3D-printed hybrid composite resins compared to conventional PMMA and bis-acrylic resins.

The poor color stability of 3D-printed provisional resins has been attributed to multiple reasons: CAD/CAM milled PMMA resins have a high polymerization rate, undergo industrial manufacturing, and have high crosslinking, thus making them dense in comparison to 3D-printed PMMA resins, which have low polymerization rates leading to poor surface integrity and color stability [14,36,37,42,55,56,57,58,59,60]. Studies reported that CAD/CAM milled and conventionally processed PMMA resins have MMA (methylmethacrylate)-based monomers that are hydrophobic, whereas HDMA (hexamethylene glycol dimethacrylate), which is the monomer used in light polymerized resins, is hydrophilic in nature. Thus, the higher polarity of 3D-printed PMMA resins could also be a reason for the poor color stability [48,61,62,63,64,65]. Studies by Atria et al. [42] and Yao et al. [45] evaluated the optical properties of 3D-printed hybrid composite resins. The poor color stability could be attributed to a lack of filler particles in these resins, thus leading to an increase in surface roughness. Song et al. [44] attributed the poor color stability to the presence of an uncured layer on the 3D-printed resins. The quantity of residual monomers, high solubility, and water sorption are also additive factors that influence the color stability of 3D-printed materials [28,66].

Myagmar et al. [47] and Atrial et al. [42] tested the color stability after artificial aging by thermocycling, whereas Shin et al. [28], Song et al. [44], and Taşın et al. [48] immersed the test specimens in different staining solutions (coffee, grape juice, curry, black tea, cola, and red wine). In general, as the immersion duration increases, the extent of discoloration increases for the tested specimens. The extent of color change also varied depending upon the type of staining solution. Studies [45,58,59,60,61] have shown that the application of surface glaze/sealant materials significantly improves the color stability and decreases the surface roughness of 3D-printed materials.

Water sorption by acrylic resins can affect the dimensional stability and can lead to failure of the prosthesis [67,68,69], whereas a high solubility of acrylic resins can lead to the presence of more unreacted monomers, which can adversely affect oral tissues. Thus, for a material to be successful, it should have minimal water sorption and solubility [70]. Two studies evaluated the sorption and solubility of 3D-printed provisional resins [28,44]. They reported that the water sorption and solubility of 3D-printed PMMA and photopolymer provisional resins were higher than CAD/CAM milled PMMA and conventional bis-acrylic resins, while the water sorption is less than in conventional PMMA provisional resins. Perea-Lowery et al. [71] and Berli et al. [68] correlated the high water sorption and solubility of 3D-printed resins to the polymerization technique. The 3D-printed materials are printed in layers, and water can enter in these layers, causing movement in the polymer chains, which can cause dimensional changes. In addition to this, the presence of free monomers in 3D-printed materials due to the low polymerization degree increases the water sorption [68,71,72].

### 4.2. Mechanical Properties

Mechanical properties discussed in the articles included in this systematic review and meta-analysis are fracture strength, microhardness, surface roughness, wear resistance, flexural strength, elastic modulus, peak stress, toughness, and resilience.

Fracture strength, flexural strength, peak stress, elastic modulus, and wear resistance are some of the mechanical properties which were found to be better for 3D-printed resins when compared to conventional and CAD/CAM milled provisional materials.

Three-dimensionally printed materials are fabricated by a layering technique; thus, there is a chemical bond between the layers [38]. The technique of fabrication affects the mechanical properties of 3D-printed resins. The authors of [38,73] reported that orientation during printing affects the mechanical properties. Vertical build orientation causes layers to be deposited perpendicular to the direction of the load application. So, these materials display superior mechanical properties compared to those printed in horizontal orientation (as layer deposition is parallel to load direction). The layer thickness during the printing process also effects the mechanical properties of these materials. Ibrahim et al. [38] and Tahayeri et al. [37] stated that the lower the layer thickness of printing is, the more layer-to-layer interfaces that will be available; thus, each layer will be polymerized in a better way, which will increase the mechanical properties of these materials. After fabrication, 3D-printed materials are subjected to post-curing, which increases the degree of conversion, thus leading to lower residual monomers and increased mechanical properties [36,41]. Park et al. [26] and Mayer et al. [50] reported that 3D-printed provisional resins contain multiple different methacrylate resins and further additives. This difference in composition can be the reason for their superior wear resistance properties.

In conventional provisional resins, which are mixed manually or by using automixing units, there are high chances of incorporating air bubbles and porosities, which can be a reason for their poor mechanical properties [16,39]. Studies reported inferior mechanical properties of CAD/CAM milled provisional resins compared to 3D-printed resins. Monomer release from PMMA Blank after aging [55], the presence of fine grooves and lines on the surface of milled resins (due to milling process) [37,74], and the presence of higher weight percentages of carbon and oxygen (representing organic part) in CAD/CAM milled provisional resins [38] can be some of the possible reasons for such behavior. On the contrary, the shrinkage of specimens during the building and post-curing processes can be a reason why few studies reported the poor mechanical properties of 3D-printed resins compared to others [36,38,75].

Toughness, resilience, and microhardness are some of the mechanical properties that are poor for 3D-printed composite-based resins compared to CAD/CAM milled PMMA resins. For long-term provisional restorations, the resiliency should be higher to avoid failures. The dense cross-linking and homogenous structure of CAD/CAM milled PMMA resins make them less prone to hydrolytic degradation when compared to conventional and 3D-printed resins [30]. In addition, the difference in composition and manufacturing technique [29,36,49,53] are some of the causes for 3D-printed resins to have these properties inferior to other tested groups.

Studies have shown contrasting results when comparing the surface roughness of 3D-printed materials and other provisional materials. Atria et al. [15] reported high surface roughness of 3D-printed hybrid resins compared to conventional and CAD/CAM-printed PMMA resins. They stated that while printing these resins factors such as curing time, orientation, and the post-curing process may play an important role. In addition to that, they used unfilled 3D resins. Contrary to this, Taşın et al. [48] found that 3D-printed hybrid resins have less surface roughness when compared to conventional and CAD/CAM PMMA resins. They stated that due to the milling and polishing process, there could be additional surface defects that can increase the surface roughness. In general, it can be stated that the surface roughness of 3D-printed resins is affected by the composition of tested resin and printing orientation [47].

This systematic review employed a comprehensive search strategy, and independent assessments of the reviewers were used during article selection to avoid bias. These are the highlights of this review. All the articles discussing the physical and mechanical properties of 3D-printed provisional materials were evaluated to ensure that no relevant article is missed.

### 4.3. Limitations

Studies included in this systematic review had medium-to-high-quality methodologies, but the risk of bias was high. High heterogeneity was observed in all meta-analyses, and most of the meta-analyses had contributions from two studies only. Most of the pooled estimates showed inconclusive results. Thus, more studies with uniformity in material and measurement techniques are needed to make conclusive statements from meta-analysis regarding the physical and mechanical properties of 3D-printed provisional resin materials. This systematic review and meta-analysis focused only on physical and mechanical properties. However, there are other parameters, such as accuracy, dimensional stability, marginal adaptation, internal adaptation, etc., which play essential roles in decision making while selecting the best material to be used for provisionalization of crowns and FDPs. Further systematic reviews are recommended to cover these aspects of the materials.

## 5. Conclusions

The following conclusions can be drawn from this systematic review and meta-analysis:

- When compared to conventional and CAD/CAM milled provisional resin materials, 3D-printed provisional crown and FDP resins have: (a) superior mechanical properties in terms of fracture strength, flexural strength, elastic modulus, peak stress, and wear resistance; (b) inferior mechanical properties in terms of toughness, resilience, and microhardness; (c) contrasting results in terms of surface roughness; and (d) inferior physical properties in terms of color stability, water sorption, and solubility.
- In vitro studies should follow blinding protocols to avoid bias.
- Three-dimensionally printed provisional crowns and FDP materials can be used as an alternative to conventional and CAD/CAM milled long-term provisional materials.

## Figures and Tables

**Table 1:** Inclusion and exclusion criteria.

Inclusion Criteria	Exclusion Criteria
Literature in English language	Literature in a language other than English
Human clinical studies	Animal studies
In vitro studies	Letters to the editor, case reports, technical reports, cadaver studies, dissertations, incomplete trials, unpublished abstracts, reports, commentaries, and review papers.
Studies comparing the physical properties of the 3D-printed provisional crowns and fixed dental prosthesis (FDP) materials with other materials and methods used for the fabrication of provisional crowns and FDP.	Studies comparing properties other than physical and mechanical properties.
Studies comparing mechanical properties of 3D-printed provisional crowns and FPD materials with other materials and methods used for the fabrication of provisional crowns and FPD.	Studies discussing properties of only 3D-printed provisional materials but do not compare them with other types of provisional materials
	Studies comparing accuracy, marginal, and internal adaptation of 3D-printed provisional materials with other types of provisional materials.
	Studies discussing effects of various 3D-printing parameters (printing orientation, resin color setting, layer thickness, degree of conversion, etc.) on mechanical properties and accuracy of 3D-printed crown and bridge provisional restorative material.
	Studies discussing materials under trial

**Table 2:** Search terms and strategy for the electronic databases.

Database	Combination of Search Terms and Strategy	Number of Titles
MEDLINE-PubMed	((“dental restoration, temporary”[MeSH Terms] OR “Tooth Crown”[MeSH Terms] OR “Dental Prosthesis”[MeSH Terms] OR “crowns”[MeSH Terms] OR “denture, partial, fixed”[MeSH Terms] OR “denture, partial, temporary”[MeSH Terms] OR “dental prosthesis, implant supported”[MeSH Terms] OR “Crown and Bridge materials”[Title/Abstract] OR “provisional dental restoration”[Title/Abstract] OR “provisional crown”[Title/Abstract] OR “provisional fixed partial denture”[Title/Abstract] OR “provisional resin”[Title/Abstract] OR “Provisional dental materials”[Title/Abstract] OR “provisional restorations”[Title/Abstract] OR “interim restoration”[Title/Abstract] OR “interim crown”[Title/Abstract] OR “interim resin”[Title/Abstract] OR “interim fixed partial denture”[Title/Abstract] OR “Temporary Crown and Bridge”[Title/Abstract] OR “temporary crown”[Title/Abstract] OR “Temporary dental restoration”[Title/Abstract]) AND “english”[Language] AND ((“printing, three dimensional”[MeSH Terms] OR “Stereolithography”[MeSH Terms] OR “3d print *”[Title/Abstract] OR “3d print*”[Title/Abstract] OR “Rapid prototyping”[Title/Abstract] OR “additive manufactur *”[Title/Abstract]) AND “english”[Language]) AND ((“Computer-Aided Design”[MeSH Terms] OR “polymethyl methacrylate”[MeSH Terms] OR “bisphenol a-glycidyl methacrylate”[MeSH Terms] OR “computer-aided manufacturing”[Title/Abstract] OR “Computer-Assisted Designing”[Title/Abstract] OR “Computer-Assisted manufacturing”[Title/Abstract] OR “Computer-Assisted Milling”[Title/Abstract] OR “cad cam”[Title/Abstract] OR “cad cam”[Title/Abstract] OR “Subtractive manufacturing”[Title/Abstract] OR “PEMA”[Title/Abstract] OR “bis-acryl”[Title/Abstract] OR “interim resin”[Title/Abstract] OR “provisional resin”[Title/Abstract] OR “Bis-GMA”[Title/Abstract] OR “methacrylate polymethyl”[Title/Abstract] OR “poly methyl methacrylate”[Title/Abstract] OR “PMMA”[Title/Abstract] OR “Polymethylmethacrylate”[Title/Abstract]) AND “english”[Language]) AND ((“Physical Phenomena”[MeSH Terms] OR “mechanical phenomena”[MeSH Terms] OR “stress, mechanical”[MeSH Terms] OR “Mechanical Tests”[MeSH Terms] OR “Flexural Strength”[MeSH Terms] OR “elasticity”[MeSH Terms] OR “elastic modulus”[MeSH Terms] OR “compressive strength”[MeSH Terms] OR “Tensile Strength”[MeSH Terms] OR “Shear strength”[MeSH Terms] OR “hardness”[MeSH Terms] OR “Hardness Tests”[MeSH Terms] OR “Dental Restoration Wear”[MeSH Terms] OR “solubility”[MeSH Terms] OR “color”[MeSH Terms] OR “Optical Phenomena”[MeSH Terms] OR “viscosity”[MeSH Terms] OR “Physical properties”[Title/Abstract] OR “Physical processes”[Title/Abstract] OR “Mechanical properties”[Title/Abstract] OR “Mechanical processes”[Title/Abstract] OR “fracture strength”[Title/Abstract] OR “Fracture resistance”[Title/Abstract] OR “fracture toughness”[Title/Abstract] OR “fracture load”[Title/Abstract] OR “Flexural Strength”[Title/Abstract] OR “Biaxial flexural strength”[Title/Abstract] OR “Yield strength”[Title/Abstract] OR “Fatigue strength”[Title/Abstract] OR “fatigue test”[Title/Abstract] OR “peak stress”[Title/Abstract] OR “Ultimate Tensile Strength Test”[Title/Abstract] OR “Shear Bond Strength”[Title/Abstract] OR “Elastic strength”[Title/Abstract] OR “Microhardness”[Title/Abstract] OR “wear resistance”[Title/Abstract] OR “surface wear”[Title/Abstract] OR “surface roughness”[Title/Abstract] OR “Texture analysis”[Title/Abstract] OR “water sorption”[Title/Abstract] OR “color tone”[Title/Abstract] OR “color masking”[Title/Abstract] OR “Translucency”[Title/Abstract] OR “Optical properties”[Title/Abstract] OR “Color Stability”[Title/Abstract] OR “Translucency”[Title/Abstract] OR “Color Change”[Title/Abstract] OR (“tarnish”[All Fields] OR “tarnishes”[All Fields] OR “tarnishing”[All Fields]) OR “corrosion”[Title/Abstract] OR “Creep”[Title/Abstract] OR “flow”[Title/Abstract] OR “Abrasion”[Title/Abstract] OR “Abrasion resistance”[Title/Abstract] OR “Brittleness”[Title/Abstract] OR “Toughness”[Title/Abstract] OR “Flexibility”[Title/Abstract]) AND “english”[Language])) AND (english[Filter])	132
Scopus	(“dental restoration, temporary” OR “Tooth Crown” OR “Dental Prosthesis” OR “crowns” OR “denture, partial, fixed” OR “denture, partial, temporary” OR “dental prosthesis, implant supported” OR “Crown and Bridge materials” OR “provisional dental restoration” OR “provisional crown” OR “provisional fixed partial denture” OR “provisional resin” OR “Provisional dental materials” OR “provisional restorations” OR “interim restoration” OR “interim crown” OR “interim resin” OR “interim fixed partial denture” OR “Temporary Crown and Bridge” OR “temporary crown” OR “Temporary dental restoration”) AND (“printing, three dimensional” OR “Stereolithography” OR “3d print *” OR “3d print *” OR “Rapid prototyping” OR “additive manufactur *”) AND (“Computer-Aided Design” OR “polymethyl methacrylate” OR “bisphenol a-glycidyl methacrylate” OR “computer-aided manufacturing” OR “Computer-Assisted Designing” OR “Computer-Assisted manufacturing” OR “Computer-Assisted Milling” OR “cad cam” OR “cad cam” OR “Subtractive manufacturing” OR “PEMA” OR “bis-acryl” OR “interim resin” OR “provisional resin” OR “Bis-GMA” OR “methacrylate polymethyl” OR “poly methyl methacrylate” OR “PMMA” OR “Polymethylmethacrylate”) AND (“Physical Phenomena” OR “mechanical phenomena” OR “stress, mechanical” OR “Mechanical Tests” OR “Flexural Strength” OR “elasticity” OR “elastic modulus” OR “compressive strength” OR “Tensile Strength” OR “Shear strength” OR “hardness” OR “Hardness Tests” OR “Dental Restoration Wear” OR “solubility” OR “color” OR “Optical Phenomena” OR “viscosity” OR “Physical properties” OR “Physical processes” OR “Mechanical properties” OR “Mechanical processes” OR “fracture strength” OR “Fracture resistance” OR “fracture toughness” OR “fracture load” OR “Flexural Strength” OR “Biaxial flexural strength” OR “Yield strength” OR “Fatigue strength” OR “fatigue test” OR “peak stress” OR “Ultimate Tensile Strength Test” OR “Shear Bond Strength” OR “Elastic strength” OR “Microhardness” OR “wear resistance” OR “surface wear” OR “surface roughness” OR “Texture analysis” OR “water sorption” OR “color tone” OR “color masking” OR “Translucency” OR “Optical properties” OR “Color Stability” OR “Translucency” OR “Color Change” OR tarnish * OR “corrosion” OR creep OR flow OR abrasion OR “Abrasion resistance” OR brittleness OR toughness OR flexibility) AND (LIMIT-TO (DOCTYPE, “ar”) OR LIMIT-TO (DOCTYPE, “cp”)) AND (LIMIT-TO (SUBJAREA, “DENT”)) AND (LIMIT-TO (LANGUAGE, “English”)) AND (LIMIT-TO (SRCTYPE, “j”) OR LIMIT-TO (SRCTYPE, “p”))	642
Web of Sciences (Core collection)	#1 (P) (TS = (“dental restoration, temporary” OR “Tooth Crown” OR “Dental Prosthesis” OR “crowns” OR “denture, partial, fixed” OR “denture, partial, temporary” OR “dental prosthesis, implant supported” OR “Crown and Bridge materials” OR “provisional dental restoration” OR “provisional crown” OR “provisional fixed partial denture” OR “provisional resin” OR “Provisional dental materials” OR “provisional restorations” OR “interim restoration” OR “interim crown” OR “interim resin” OR “interim fixed partial denture” OR “Temporary Crown and Bridge” OR “temporary crown” OR “Temporary dental restoration”)) AND LANGUAGE: (English) Indexes=SCI-EXPANDED, SSCI, A&HCI, CPCI-S, CPCI-SSH, ESCI, CCR-EXPANDED, IC Timespan = All years #2 (I) (TS = (“printing, three dimensional” OR “Stereolithography” OR “3d print *” OR “3d print *” OR “Rapid prototyping” OR “additive manufactur *”)) AND LANGUAGE: (English) Indexes=SCI-EXPANDED, SSCI, A&HCI, CPCI-S, CPCI-SSH, ESCI, CCR-EXPANDED, IC Timespan = All years #3 (C) (TS = (“Computer-Aided Design” OR “polymethyl methacrylate” OR “bisphenol a-glycidyl methacrylate” OR “computer-aided manufacturing” OR “Computer-Assisted Designing” OR “Computer-Assisted manufacturing” OR “Computer-Assisted Milling” OR “cad cam” OR “cad cam” OR “Subtractive manufacturing” OR “PEMA”OR “bis-acryl” OR “interim resin” OR “provisional resin”OR “Bis-GMA” OR “methacrylate polymethyl” OR “poly methyl methacrylate” OR “PMMA” OR “Polymethylmethacrylate”)) AND LANGUAGE: (English) Indexes = SCI-EXPANDED, SSCI, A&HCI, CPCI-S, CPCI-SSH, ESCI, CCR-EXPANDED, IC Timespan = All years #4 (O) (TS = (“Physical Phenomena” OR “mechanical phenomena” OR “stress, mechanical” OR “Mechanical Tests” OR “Flexural Strength” OR “elasticity” OR “elastic modulus” OR “compressive strength” OR “Tensile Strength” OR “Shear strength” OR “hardness” OR “Hardness Tests” OR “Dental Restoration Wear” OR “solubility” OR “color” OR “Optical Phenomena” OR “viscosity” OR “Physical properties” OR “Physical processes” OR “Mechanical properties” OR “Mechanical processes” OR “fracture strength” OR “Fracture resistance” OR “fracture toughness” OR “fracture load” OR “Flexural Strength” OR “Biaxial flexural strength” OR “Yield strength” OR “Fatigue strength” OR “fatigue test” OR “peak stress” OR “Ultimate Tensile Strength Test” OR “Shear Bond Strength” OR “Elastic strength” OR “Microhardness” OR “wear resistance” OR “surface wear” OR “surface roughness” OR “Texture analysis” OR “water sorption” OR “color tone” OR “color masking” OR “Translucency” OR “Optical properties” OR “Color Stability” OR “Translucency” OR “Color Change” OR tarnish* OR “corrosion” OR Creep OR flow OR Abrasion OR “Abrasion resistance” OR Brittleness OR Toughness OR Flexibility)) AND LANGUAGE: (English) Indexes = SCI-EXPANDED, SSCI, A&HCI, CPCI-S, CPCI-SSH, ESCI, CCR-EXPANDED, IC Timespan = All years #4 AND #3 AND #2 AND #1 Indexes = SCI-EXPANDED, SSCI, A&HCI, CPCI-S, CPCI-SSH, ESCI, CCR-EXPANDED, IC Timespan = All years and English (Languages)	33
Cochrane Library	#1 MeSH descriptor: [Dental Restoration, Temporary] explode all trees #2 MeSH descriptor: [Tooth Crown] explode all trees #3 MeSH descriptor: [Dental Prosthesis] explode all trees #4 MeSH descriptor: [Crowns] explode all trees #5 MeSH descriptor: [Denture, Partial, Fixed] explode all trees #6 MeSH descriptor: [Denture, Partial, Temporary] explode all trees #7 MeSH descriptor: [Dental Prosthesis, Implant-Supported] explode all trees #8 Crown and Bridge material * #9 provisional dental restoration #10 provisional crown #11 provisional fixed partial denture #12 provisional resin #13 Provisional Crown and Bridge #14 Provisional Crown and Bridge material * #15 Provisional dental material * #16 provisional restoration * #17 Provisional Implant-Supported Fixed Dental Prosthes * #18 interim restoration #19 interim crown #20 interim resin #21 interim fixed partial denture #22 Temporary Crown and Bridge #23 temporary crown #24 Temporary dental restoration #25 MeSH descriptor: [Printing, Three-Dimensional] explode all trees #26 MeSH descriptor: [Stereolithography] explode all trees #27 3D print * #28 3D-print * #29 Rapid prototyping #30 #30 Additive manufactur * #31 MeSH descriptor: [Computer-Aided Design] explode all trees #32 MeSH descriptor: [Polymethyl Methacrylate] explode all trees #33 MeSH descriptor: [Bisphenol A-Glycidyl Methacrylate] explode all trees #34 computer-aided manufactur * #35 Computer-Assisted Design * #36 Computer-Assisted manufactur * #37 Computer-Assisted Mill * #38 CAD-CAM #39 CAD CAM #40 Subtractive manufactur * #41 Conventional cur * #42 Conventional polymeriz * #43 PEMA #44 bis-acryl #45 interim resin #46 provisional resin #47 Bis-GMA #48 Methacrylate, Polymethyl #49 Poly(methyl methacrylate) #50 PMMA #51 Polymethylmethacrylate #52 MeSH descriptor: [Physical Phenomena] explode all trees #53 MeSH descriptor: [Mechanical Phenomena] explode all trees #54 MeSH descriptor: [Stress, Mechanical] explode all trees #55 MeSH descriptor: [Mechanical Tests] explode all trees #56 MeSH descriptor: [Flexural Strength] explode all trees #57 MeSH descriptor: [Elasticity] explode all trees #58 MeSH descriptor: [Elastic Modulus] explode all trees #59 MeSH descriptor: [Compressive Strength] explode all trees #60 MeSH descriptor: [Tensile Strength] explode all trees #61 MeSH descriptor: [Shear Strength] explode all trees #62 MeSH descriptor: [Hardness] explode all trees #63 MeSH descriptor: [Hardness Tests] explode all trees #64 MeSH descriptor: [Dental Restoration Wear] explode all trees #65 MeSH descriptor: [Solubility] explode all trees #66 MeSH descriptor: [Color] explode all trees #67 MeSH descriptor: [Optical Phenomena] explode all trees #68 MeSH descriptor: [Viscosity] explode all trees #69 Physical propert * #70 Physical processe * #71 Mechanical propert * #72 Mechanical processe * #73 fracture strength #74 Fracture resistance #75 fracture toughness #76 fracture load #77 Flexural Strength #78 Biaxial flexural strength #79 Yield strength #80 Fatigue strength #81 fatigue test #82 peak stress #83 Ultimate Tensile Strength Test #84 Shear Bond Strength #85 Elastic strength #86 Microhardness #87 wear resistance #88 surface wear #89 surface roughness #90 Texture analysis #91 water sorption #92 color tone #93 color masking #94 Translucency #95 Optical propert * #96 Color Stability #97 Translucency #98 Color Change #99 Tarnish #100 corrosion #101 Creep #102 flow #103 Abrasion #104 Abrasion resistance #105 Brittleness #106 Toughness #107 Flexibility #108 #1 OR #2 OR #3 OR #4 OR #5 OR #6 OR #7 OR #8 OR #9 OR #10 OR #11 OR #12 OR #13 OR #14 OR #15 OR #16 OR #17 OR #18 OR #19 OR #20 OR #21 OR #22 OR #23 OR #24 #109 #25 OR #26 OR #27 OR #28 OR #29 OR 30 #110 #31 OR #32 OR #33 OR #34 OR #35 OR #36 OR #37 OR #38 OR #39 OR #40 OR #41 OR #42 OR #43 OR #44 OR #45 OR #46 OR #47 OR #48 OR #49 OR #50 OR #51 #111 #52 OR #53 OR #54 OR #55 OR #56 OR #57 OR #58 OR #59 OR #60 OR #61 OR #62 OR #63 OR #64 OR #65 OR #66 OR #67 OR #68 OR #69 OR #70 OR #71 OR #72 OR #73 OR #74 OR #75 OR #76 OR #77 OR #78 OR #79 OR #80 OR #81 OR #82 OR #83 OR #84 OR #85 OR #86 OR #87 OR #88 OR #89 OR #90 OR #91 OR #92 OR #93 OR #94 OR #95 OR #96 OR #97 OR #98 OR #99 OR #100 OR #101 OR #102 OR #103 OR #104 OR #105 OR #106 OR #107 #112 #108 AND #109 AND #110 AND #111	89

*: Truncation, P: Population, I: Intervention, C: Comparator, O: Outcome.

## Data Availability

The data that support the findings of this study are available from the corresponding author upon reasonable request.

## Supplementary Materials

The following supporting information can be downloaded at: https://www.mdpi.com/article/10.3390/polym14132691/s1. Table S1: PRISMA 2020 Main Checklist.
